# A Bone Marrow–Mimetic Hydrogel Enables Dual‐Phase Hemostasis and Vascularized Osteogenesis for Cranial Defects

**DOI:** 10.1002/advs.75590

**Published:** 2026-05-08

**Authors:** Lingbin Che, Donghong Li, Huan Zhang, Tingting Xu, Juhan Li, Xuanzhou Chen, Louis D. Zhang, Shuguang Wang, Dianwen Song, Dongyong Sha

**Affiliations:** ^1^ Department of Orthopedics, Shanghai General Hospital Shanghai Jiao Tong University School of Medicine Shanghai China; ^2^ College of Biological Science and Medical Engineering Donghua University Shanghai Shanghai China; ^3^ Department of Emergency Medicine The Affiliated Hospital of Xuzhou Medical University, School of Second Clinical Medicine of Xuzhou Medical University Xuzhou Jiangsu China; ^4^ School of Electrical and Computer Engineering Georgia Institute of Technology Atlanta Georgia USA; ^5^ Power Dream America, Inc. Peachtree Corners Georgia USA; ^6^ Trauma Center, Shanghai General Hospital Shanghai Jiao Tong University School of Medicine Shanghai China

**Keywords:** biomimetic hydrogel, cranial regeneration, fat extract, osteogenesis and angiogenesis

## Abstract

Critical‐sized cranial defects present two sequential clinical challenges. These include an acute need for rapid hemostasis and a long‐term requirement for vascularized bone regeneration. Current implants fail to address these sequential demands. To overcome this limitation, a bone marrow‐mimetic composite hydrogel (FE‐PDA@Fib/Gel‐TG) is engineered. This system integrates transglutaminase crosslinked gelatin, rigid polydopamine‐coated hydroxyapatite/poly(L‐lactic acid) (HAp/PLLA) short fibers, and cell‐free fat extract (FE). These components together recapitulate key biochemical and biomechanical features of native bone marrow. The hierarchically designed scaffold immediately achieves hemostasis through fiber‐mediated mechanical sealing and catechol‐assisted clot stabilization. Furthermore, the sustained release of FE establishes a pro‐regenerative microenvironment. This milieu significantly enhances cell recruitment, endothelial network formation, and osteogenic differentiation. It also promotes heterotypic crosstalk between endothelial and osteoprogenitor cells. Transcriptomic analyses reveal that this vascular‐bone coupling is driven by the convergent activation of VEGF/VEGFR‐PI3K‐AKT signaling pathways. In a critical‐sized calvarial defect model, the hydrogel actively steers macrophage polarization toward an anti‐inflammatory phenotype. Consequently, it induces the robust regeneration of morphologically mature, highly vascularized bone tissue. By successfully coupling rapid hemostatic control with spatiotemporally programmed osteo‐angiogenesis, this multifunctional biomimetic platform represents a highly translatable advancement for effective cranial defect repair.

## Graphical abstract

A biomimetic, fiber‐reinforced hydrogel (FE‐PDA@Fib/Gel‐TG) that enables dual‐phase cranial defect repair. The system provides: instant hemostasis in the early stage, and sustained co‐delivery of pro‐angiogenic and osteogenic signals for synergistic vascularized bone regeneration.

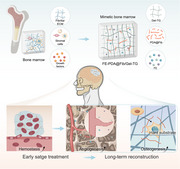

## 1 Introduction

**FIGURE 1:**
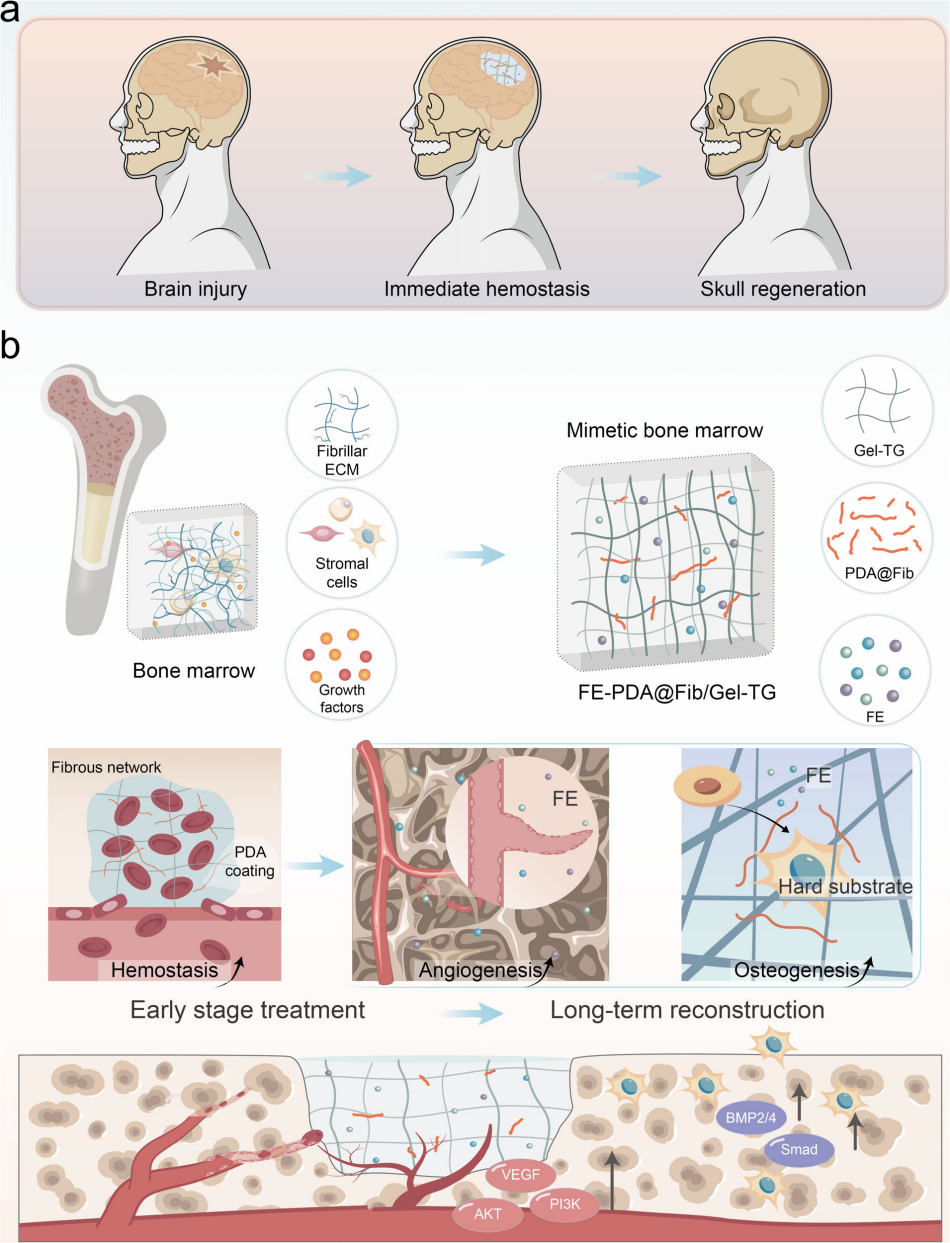
Biomimetic design and therapeutic concept of the FE‐PDA@Fib/Gel‐TG hydrogel for cranial defect repair. (a) This schematic illustrates the integrated therapeutic mechanism. Following implantation, the materials provide rapid hemostasis at the diploe and subsequently orchestrates coupled angiogenesis and osteogenesis. (b) The construction of the composite hydrogel recapitulating key compositional and structural features of native bone marrow. The FE‐PDA@Fib/Gel‐TG composite achieves efficient hemostasis during the early stage. It ultimately facilitates long term and functional cranial bone regeneration.

Critical‐sized cranial defects, commonly caused by tumor resection or physical trauma, represent a clinically challenging condition that may compromise intracranial integrity and expose underlying tissues [1, 2, 3]. Clinically used implants such as titanium mesh and polyetheretherketone (PEEK) mainly provide structural support but are biologically inert; they neither direct new bone regeneration nor support vascular growth, both of which are indispensable for repair of the vascularized cranial bone [4, 5]. Notably, managing bleeding from the vascular‐rich diploic bone often necessitates the use of auxiliary agents like bone wax, which, however, is known to hinder healing at the bone interface [6]. Successful cranial reconstruction therefore requires more than morphological restoration of the defect: the repaired region must re‐establish an intact, mechanically competent cranial vault to protect the brain while simultaneously achieving functional tissue regeneration. This imposes two sequential requirements on any repair strategy. First, hemorrhage from the diploe must be rapidly controlled within the early post‐injury golden window for prevent hematoma formation and secondary damage [7, 8]. Second, the implant should act as a bioactive “regenerative engine” that guides long‐term osteogenesis and angiogenesis [9, 10], ultimately enabling stable, functional restoration of the cranial cavity (Figure 1a).

To enhance implant bioactivity, a widely adopted strategy to enhance implant bioactivity involves the exogenous delivery of single growth factors such as bone morphogenetic protein‐2 (BMP‐2) or vascular endothelial growth factor (VEGF) [11, 12]. However, these strategies suffer from difficult dose control and short half‐life, and may induce adverse effects, including heterotopic ossification and excessive inflammation [13, 14]. Although combining multiple recombinant factors could better mimic the native microenvironment, their complex formation and high cost impede clinical translation [15]. In this context, cell‐free fat extract (FE) has emerged as an attractive, low‐cost alternative. FE contains a broad spectrum of growth factors and functional proteins with low immunogenicity and robust bioactivity, providing a more physiologically relevant cue set than single‐factor delivery [16, 17, 18, 19, 20, 21]. This concept is consistent with a paradigm shift from simply supplying exogenous factors to reconstructing a functional regenerative microenvironment. Bone marrow aspirate is a clinically available example of such an endogenous repair system, providing a complex and balanced mixture of cell and humoral factors that support autonomous tissue regeneration [22, 23]. Structurally, bone marrow involves some key elements (i.e., a fibrous extracellular matrix (ECM), diverse signaling molecules and growth factors, and stromal cells), which together regulate both hematopoiesis and osteogenesis [24, 25, 26]. The fate of stromal cells is further modulated by ECM stiffness and microarchitecture [27]. In contrast to long bone and the axial skeleton, which possess a central marrow cavity rich in vasculature and osteoprogenitor cells, cranial bone exhibit inherently limited regenerative capacity [28, 29, 30]. Thus, for an effective repair strategy, it is highly desirable to construct a bone marrow‐mimetic microenvironment that recapitulates both the biochemical composition and multiscale structural cues of native marrow, while also providing matched degradation and mechanical compatibility with the skull [31, 32, 33, 34, 35, 36].

In this study, we report a marrow‐mimetic composite hydrogel, FE‐PDA@Fib/Gel‐TG, rationally designed to couple rapid hemostasis with sustained osteo‐angiogenic regeneration in cranial defects (Figure 1b). Specifically, an enzymatically cross‐linked gelatin network (Gel‐TG) serves as a cell‐permissive, biodegradable matrix into which short, rigid fibers of hydroxyapatite/poly(L‐lactic acid) (HAp/PLLA, Fib) are embedded. Electrospun HAp/PLLA mats were fragmented by ultrasonication and conformally coated with polydopamine (PDA), yielding hydrophilic, high‐modulus, mineralized filaments bearing catechol and charged groups. After implantation, the PDA@Fib phase entangles within the hydrogel and against the trabecular diploe to form a mechanically robust barrier, while the adhesive polyphenolic surface chemistry confers strong physical and chemical hemostatic activity, enabling efficient sealing of cancellous bone bleeding in the early post‐injury stage [37, 38]. In parallel, cell‐free fat extract (FE) is homogeneously loaded into Gel‐TG and released in a sustained manner, recreating the multicomponent growth‐factor milieu of bone marrow and reprogramming the defect into a pro‐angiogenic, pro‐osteogenic niche. By integrating endogenous factor‐rich FE, a gelatinous ECM analogue, and osteoconductive PDA‐coated HAp/PLLA reinforcement, FE‐PDA@Fib/Gel‐TG bridges the transition from acute damage control to long‐term regeneration, ultimately supporting vascularized, structurally competent cranial bone repair.

## 2 Results and Discussion

### 2.1 Fabrication and Characterization of Fibrous Reinforced Hydrogel Composites

**FIGURE 2:**
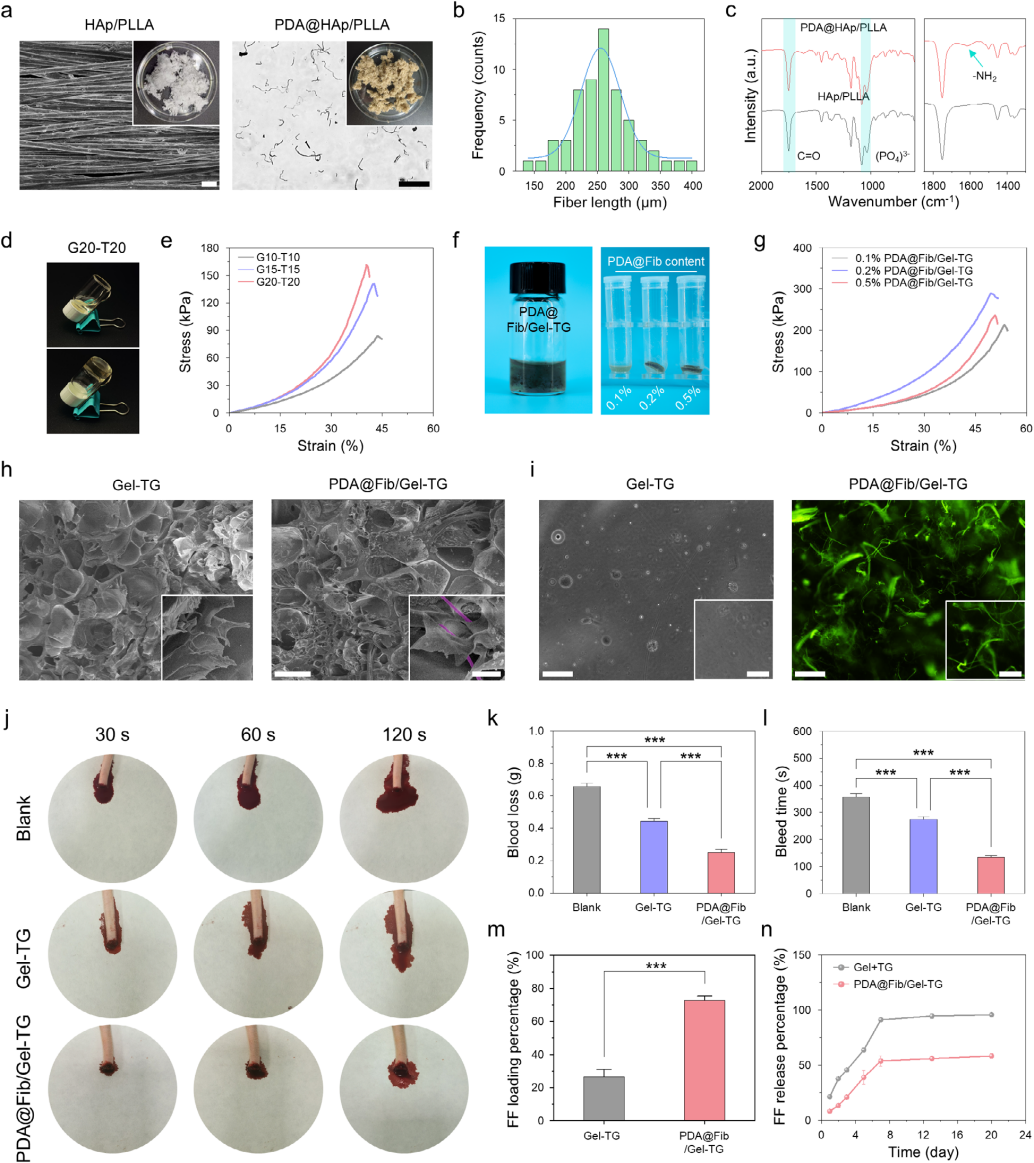
Fabrication and characterization of PDA@Fib/Gel‐TG composite hydrogels. (a) SEM image of aligned HAp/PLLA fibers (left; inset: macroscopic view of fragmented HAp/PLLA short fibers) and optical micrograph of PDA@HAp/PLLA short fibers (right; inset: macroscopic view of PDA@HAp/PLLA short fibers). (b) Length distribution of the short fibers. (c) FTIR spectra of HAp/PLLA and PDA@HAp/PLLA short fibers. (d) Gelation behavior of Gel‐TG hydrogel demonstrated by the vial inversion test. (e) Representative compressive stress‐strain curves of Gel‐TG hydrogel at the crosslinking plateau stage. (f) Gelation status and macroscopic appearance of PDA@Fib/Gel‐TG composite hydrogels with different fiber loadings. (g) Representative compressive stress‐strain curves of PDA@Fib/Gel‐TG composites with different fiber contents at the crosslinking plateau stage. (h) SEM images of Gel‐TG (left) and PDA@Fib/Gel‐TG (right); PDA@Fib within the composite are pseudocolored in purple (insets: high‐magnification views). (i) Bright‐field image of Gel‐TG (left) and fluorescence micrograph of PDA@Fib/Gel‐TG (right) showing the homogeneous distribution of Co‐6–labeled short fibers within the composite (insets: high‐magnification views). (j) Photographs of tail hemorrhages in mice at different time points. (k) Volume of blood loss in the tail of mice among different treatment groups (*n* = 3). (l) Hemostasis time in the tail of mice among different treatment groups (*n* = 3). (m) Adsorption/fixation efficiency of growth factor FE on Gel‐TG and PDA@Fib/Gel‐TG (*n* = 3). (n) In vitro FE release profiles from Gel‐TG and PDA@Fib/Gel‐TG. Scale bar: 5 µm for HAp/PLLA and 100 µm for PDA@HAp/PLLA in (a); 500 µm (main image) and 10 µm (inset) in (h); 50 µm (main image) and 10 µm (inset) in (i). Data are shown as means ± SD. Statistical analysis was performed using one‐way ANOVA with Tukey's post‐hoc test. *p < 0.05, **p < 0.01, and ***p < 0.001; ns, not significant.

Substrate stiffness is a key regulator of osteogenic differentiation; cells cultured on rigid matrices preferentially commit to an osteoblastic lineage. Guided by this principle, we first engineered degradable rigid fibers based on PLLA via electrospinning (Figure S1). Scanning electron microscopy (SEM) imaging revealed that the as‐spun HAp/PLLA fibers were highly aligned and possessed a uniform diameter distribution (Figure 2a). Incorporation of HAp nanoparticles into the PLLA matrix generated microscale surface roughness, which is expected to increase specific surface area and provide additional nucleation sites for mineral deposition. To render the fibrous phase injectable and compatible with irregularly shaped defects, the continuous bundles were mechanically fragmented into short fibers [39]. The homogenized short HAp/PLLA fibers appeared as a white powder (inset, Figure 2a), which turned uniformly brown after polydopamine (PDA) deposition, indicative of successful surface coating. Optical microscopy combined with length statistics (Figure 2b) confirmed that the majority of the short fibers fell within 220–300 µm, a range appropriate for percolated reinforcement while maintaining injectability, and SEM confirms a 0.26 µm conformal polydopamine coating (Figure S2). The fragmentation step naturally loosens the original dense structural entanglement, and the polydopamine encapsulation further reduces the surface stiffness. Atomic force microscopy (AFM) visualizes these mechanical property transitions to reveal a decreased overall surface modulus compared to the original long fibers. This functional modification provides interaction sites for subsequent material integration. The final mechanical stiffness still reaches approximately 1.17 GPa (Figure S3) to ensure these rigid fibers provide mechanical anchors inside the soft gel and create a favorable microenvironment for subsequent osteogenic differentiation. The chemical composition of the fibers before and after PDA treatment was analyzed by Fourier transform infrared (FTIR) spectroscopy (Figure 2c). Both HAp/PLLA and PDA‐coated fibers (PDA@Fib) exhibited the characteristic bands of PLLA (C═O stretching at 1760 cm^−1^) and HAp (PO_4_^3−^ vibration at 1063 cm^−1^) [40, 41]. A new absorption band appeared at 1559 cm^−1^ in the PDA@Fib spectrum, corresponding to the N‐H bending vibration of primary amines (‐NH_2_) in PDA [42]. This additional band provides direct spectroscopic evidence that a PDA layer was successfully immobilized on the fiber surface. To mimic the collagenous matrix of native bone, we constructed a gelatin‐based hydrogel using transglutaminase (TGase) as a biocatalyst. TGase catalyzes the formation of ε‐(γ‐glutamyl)lysine isopeptide bonds between the γ‐carboxamide groups of glutamine and the ε‐amino groups of lysine residues (Figure S4), thereby establishing a covalently crosslinked three‐dimensional network. Unlike physically crosslinked gelatin, which readily undergoes gel‐sol transition upon heating, this chemical crosslinked network is thermally stable within the range relevant for biomedical use.

Three formulations with varying gelatin/TGase contents (G10‐T10: 10% Gel+10 U/mL TG; G15‐T15: 15% Gel+15 U/mL TG; G20‐T20: 20% Gel+20 U/mL TG) were systematically evaluated. Vial inversion tests showed that the gelation time decreased from 3 to 2 and 1.5 min with increasing precursor concentrations (Figure 2d, Figure S5a), reflecting the accelerated formation of covalent crosslinks at higher densities of reactive groups. All hydrogels maintained shape integrity over 0.5–12 h, indicating that the network is rapidly established and mechanically robust. Monitoring of the crosslinking kinetics (Figure S5b) demonstrated a gradual increase in crosslinking degree that plateaued at 6 h (G10–T10: 59.1%; G15–T15: 69.2%; G20–T20: 79.8%). The plateau is likely governed by diffusion‐limited access of TGase in the densifying network and the consumption of available reactive sites. Consistent with the crosslinking trend, compression testing demonstrated that G20–T20 possessed the highest compressive strength (27.5 kPa), which was 2.2‐ and 1.3‐fold higher than that of G10–T10 and G15–T15, respectively (Figure 2e, Figure S5c). These results confirm a positive correlation between crosslinking density and mechanical performance. To generate fibrous‐reinforced composites, PDA‐coated short fibers were incorporated into G20–T20. Introducing 0.1% and 0.5% (w/v) PDA@Fib increased gelation time to 2.0 and 3.5 min, respectively, compared to 1.5 min for the pristine hydrogel, and reduced 12‐h crosslinking degree from 79.8% to 65.5%, 61.8% and 50.1% for 0.1%, 0.2%, and 0.5% fiber loadings (Figure 2f, Figure S6a,b). This attenuated crosslinking is attributed to competitive non‐covalent interactions between gelatin and fiber surface (hydrogen bonding and electrostatic interactions), which partially sequester reactive groups and impede TGase‐mediated bond formation. Despite this reduction in covalent crosslinking, moderate fiber incorporation significantly reinforced the hydrogel. The composite containing 0.2% PDA@Fib reached an optimal compressive strength of 35.2 kPa, representing a 27.8% increase over the pure hydrogel and surpassing the 0.1% (31.3 kPa) and 0.5% (30.4 kPa) groups (Figure 2g, Figure S6c). This non‐monotonic dependence on fiber content can be rationalized as follows: at low loading (0.1%), the fiber network is below its optimal percolation level and thus underutilized as a reinforcing phase; at high loading (0.5%), fiber aggregation and inter‐fiber friction compromise dispersion homogeneity and impair stress transfer. In contrast, 0.2% PDA@Fib achieves a balance between network connectivity, matrix continuity and crosslinking, resulting in maximal reinforcement efficiency. Accordingly, G20‐T20 was designated as Gel‐TG, and the 0.2% PDA@Fib composite hydrogel was termed PDA@Fib/Gel‐TG for subsequent studies. The microstructure of hydrogels was examined by SEM after freeze‐drying. Both Gel‐TG and PDA@Fib/Gel‐TG exhibited highly porous, interconnected networks (Figure 2h). High‐magnification images of Gel‐TG showed relatively smooth pore walls, whereas clear fiber segments embedded within the pore walls were observed in PDA@Fib/Gel‐TG (inset, Figure 2h). To further elucidate the spatial distribution, HAp/PLLA fibers were labelled with coumarin 6 (Co‐6/HAp/PLLA). The fluorescence of the labeled fibers remained stable after PDA coating (Co‐6/PDA@Fib) (Figure S7). Fluorescence microscopy of the composites containing Co‐6/PDA@Fib demonstrated homogeneous dispersion of fibers throughout the hydrogel and preservation of their morphology at higher magnification (Figure 2i). As expected, no fluorescence was detected in the Gel‐TG.

Mechanical testing clearly highlighted the contribution of both the fibrous phase and PDA interfacial modification. Incorporation of unmodified HAp/PLLA short fibers into Gel‐TG (Fib/Gel‐TG) led to a 19.7% increase in Young's modulus and a 61.1% improvement in compressive strength relative to Gel‐TG, confirming the basic reinforcing role of the rigid fibers (Figure S8a–c). When the fibers were PDA‐coated (PDA@Fib/Gel‐TG), the mechanical enhancements became more pronounced: Young's modulus and compressive strength increased by 89.5% and 69.1%, respectively, compared to Gel‐TG. Relative to Fib/Gel‐TG, PDA@Fib/Gel‐TG further exhibited a 58.0% higher modulus and a 5.1% higher strength (Figure S8b,c). Additionally, rheological evaluations confirm this structural stability. All hydrogels maintain stable dynamic networks across the tested range (Figure S8d). The rheological modulus trend aligns with the macroscopic compressive data. These improvements are ascribed to the catechol/quinone chemistry of PDA, which forms covalent bonds (via Michael addition or Schiff‐base reactions) with primary amines on gelatin. This interfacial coupling increases load transfer efficiency between the stiff fibers and the soft matrix, thereby enhancing the composite's stiffness and toughness. Water uptake measurement provided additional insight into the network structure (Figure S9). The equilibrium swelling ratio of PDA@Fib/Gel‐TG (479.4%) was markedly lower than that of Gel‐TG (723.8%). This restricted swelling behavior is primarily attributed to a dual‐mechanism: the intrinsic hydrophobicity of the incorporated HAp/PLLA fibers and the augmented network crosslinking density facilitated by the PDA‐mediated interfacial bonding between the fibers and the gelatin matrix. From a functional standpoint, moderate swelling is advantageous for absorbing exudates while preventing excessive volume expansion that could disrupt clot formation or tissue integration. The hemostatic performance of the hydrogels was evaluated in a mouse tail amputation model (Figure 2j). All formulations exhibited some degree of bleeding control; however, PDA@Fib/Gel‐TG showed the best hemostatic efficacy, achieving stable hemostasis within ∼135 s and limiting blood loss to 0.25 ± 0.02 g (Figure 2k,l), significantly outperforming the non‐reinforced Gel‐TG. A commercial Gel‐foam group is also included for comparison (Figure S10). The PDA@Fib/Gel‐TG composite exhibits comparable and even slightly superior hemostatic performance relative to this commercial benchmark. This enhanced performance stems from a multifaceted synergistic effect, whereby the dense, mechanically robust fibrous network rapidly constructs a physical barrier at the bleeding site, while the controlled swelling prevents the over‐dilution of localized clotting factors; simultaneously, the catechol‐rich PDA coating further accelerates the process by promoting platelet recruitment and activation [37, 43]. Consequently, the composite maintains a high fluid‐uptake capacity while providing a stable, bioactive interface, satisfying the essential requirements for both tissue engineering scaffolds and advanced wound management systems. Enzymatic degradation studies further highlighted the stabilizing effect of the fibrous phase. Over 11 days, Gel‐TG degraded rapidly, retaining only 22.6% of its initial mass, whereas PDA@Fib/Gel‐TG maintained 44.7% residual mass (Figure S11). This slower degradation is governed by two complementary mechanisms: (i) the polymeric HAp/PLLA fibers themselves are more resistant to enzymatic digestion than the gelatin matrix; and (ii) covalent bonds between PDA quinones and gelatin amines strengthen fiber‐matrix interfaces, reducing the accessibility of degradative enzymes and retarding hydrogel erosion.

The in situ mineralization capacity is evaluated by immersion in a mineralizing solution. X‐ray diffraction (XRD) analysis identifies the resulting deposits as hydroxyapatite (HAp). This crystalline phase is confirmed by the characteristic diffraction peak at 2θ ≈ 32° [44]. This signal corresponds to the (211) crystallographic plane (Figure S12a). Before mineralization, the PDA@Fib/Gel‐TG composite displays a weak HAp signal. This originates from the HAp/PLLA fibers embedded within the hydrogel network. In contrast, the Gel‐TG group shows no initial mineral peaks. After immersion, both hydrogels exhibit a significant increase in HAp peak intensity compared to their non‐mineralized states. This indicates successful *de novo* mineral deposition. SEM imaging provides morphological evidence for these crystalline changes (Figure S12b). The PDA@Fib/Gel‐TG composite shows a higher density of apatite‐like deposits than the Gel‐TG group. These distinct mineralization patterns reflect the different features of each hydrogel. In Gel‐TG, mineralization is primarily driven by carboxyl groups on gelatin, which act as nucleation sites [45]. In PDA@Fib/Gel‐TG, mineralization is further amplified through a triple‐synergistic mechanism: (i) carboxyl groups on gelatin provide baseline nucleation sites; (ii) pre‐existing HAp within the fibers offers crystallographic templates; and (iii) the PDA coating, rich in catechol groups, chelates calcium ions and promotes heterogeneous nucleation and subsequent crystal growth. Finally, the loading and release of FE were investigated. Bicinchoninic acid assays showed that PDA@Fib/Gel‐TG achieved a markedly higher FE loading efficiency (72.8%) than Gel‐TG (26.6%) (Figure 2m). The pristine gelatin network exhibited a pronounced burst release, with 95.6% of FE released within 7 h (Figure 2n). In contrast, PDA@Fib/Gel‐TG displayed a moderated initial release (58.2% at 7 h), followed by a more sustained release phase. This transition from burst to controlled release can be ascribed to the multifactorial interactions introduced by the PDA‐coated fibers: π–π stacking and hydrogen bonding between FE and PDA, physical entrapment within the fibrous network, and reduced diffusivity in the denser composite matrix. Collectively, these results demonstrate that PDA@Fib/Gel‐TG provides a mechanically robust, mineralization‐competent and drug‐reservoir‐like environment, making it a promising candidate for bone‐related tissue engineering and hemostatic applications.

### 2.2 In Vitro Bioactivity of Composite Fiber‐Hydrogel Mimicking the Bone Marrow Microenvironment

**FIGURE 3:**
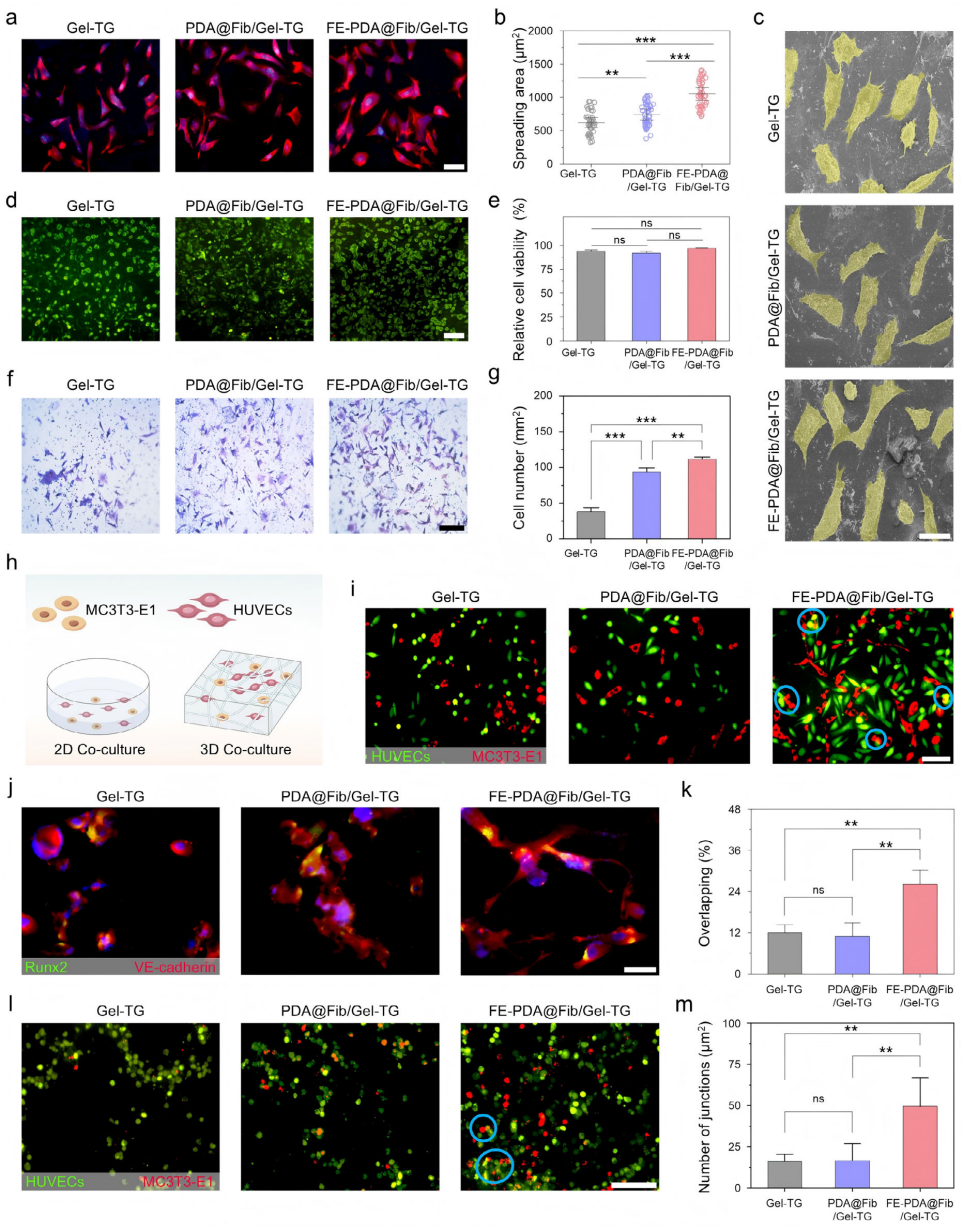
In vitro bioactivity and endothelial‐osteoblast crosstalk on engineered hydrogel substrates. (a) Cytoskeleton staining of cells cultured on different hydrogels (F‐actin, red; nuclei, blue). (b) Quantitative analysis of cell spreading area (with a total of 42 randomly selected fields analyzed). (c) SEM images of cell morphology on hydrogels; adherent cells are pseudo‐colored in yellow. (d) Live/dead fluorescence staining. (e) Relative cell viability calculated from (d) (*n* = 3). (f) Crystal violet staining showing cell adhesion and proliferation/migration. (g) Quantification of the number of migrated cells (*n* = 3). (h) Schematic of MC3T3‐E1 cells‐HUVECs connectivity in 2D and 3D microenvironments. (i) Fluorescence images of 2D co‐cultured HUVECs (green) and MC3T3‐E1 (red). (j) Double immunofluorescence staining for VE‐cadherin (red) and Runx2 (green). (k) Percentage of overlapping area of VE‐cadherin and Runx2 fluorescence signals (*n* = 3). (l) Tube‐like networks formed by HUVECs (green) co‐cultured with MC3T3‐E1 cells (red) within the hydrogels; blue circles highlight representative junctions. (m) Quantitative analysis of the number of junctions in the network shown in (l) (*n* = 3). Scale bars: 50 µm in (a), 100 µm in (c), 250 µm in (d), 50 µm in (f), 50 µm in (i), 100 µm in (j), and 50 µm in (l). Data are shown as means ± SD. Statistical analysis was performed using one‐way ANOVA with Tukey's post‐hoc test. *p < 0.05, **p < 0.01, and ***p < 0.001; ns, not significant.

To evaluate whether the composite hydrogels could recapitulate certain biophysical aspects of the bone marrow niche, we first examined the morphology of co‐cultured human umbilical vein endothelial cells (HUVECs) and murine pre‐osteoblast cell line (MC3T3‐E1) cells on the different substrates. Fluorescence staining (Figure 3a) showed that cells in all groups were able to adhere and spread; however, quantitative analysis revealed dependence of cell spreading on scaffold composition. The PDA@Fib/Gel‐TG substrates significantly increased the projected cell area relative to Gel‐TG, and the FE‐PDA@Fib/Gel‐TG group exhibited the most pronounced spreading, with average cell areas 1.7‐fold and 1.4‐fold higher than those on Gel‐TG and PDA@Fib/Gel‐TG, respectively (Figure 3b). SEM imaging further corroborated these observations, showing well‐spread cells with abundant filopodia and lamellipodia on the fiber‐reinforced composites, particularly on FE‐functionalized surfaces (Figure 3c). These data suggest that the stiffer, topographically complex PDA@Fib/Gel‐TG matrix, potentially in synergy with FE‐related biochemical interactions, provides favorable mechanical and structural properties that promote cytoskeletal organization and cell anchorage.

Cell survival and proliferation on the hydrogels were then evaluated. Live/dead staining demonstrated excellent cytocompatibility of all materials, with over 90% of cells remaining viable and only fewer dead cells were observed (Figure 3d,e). Consistently, MTT assays (Figure S13) showed sustained metabolic activity over time for all groups, with the FE‐PDA@Fib/Gel‐TG scaffold supporting the highest metabolic activity. This enhancement may be due to the mitogenic effect of FE combined with the improved mechanical microenvironment of the composite hydrogel. Collectively, these results confirm that the FE‐PDA@Fib/Gel‐TG system is highly cytocompatible and capable of actively supporting endothelial and osteoprogenitor cell growth. To probe cell recruitment potential of the scaffolds, Transwell migration experiments were performed. As shown in Figure 3f, substantially more cells migrated toward the FE‐PDA@Fib/Gel‐TG group than toward the non‐functionalized hydrogels. Quantitative analysis (Figure 3g) revealed 2.9‐fold and 1.2‐fold increases in the number of migrated cells compared with Gel‐TG and PDA@Fib/Gel‐TG, respectively, indicating that FE immobilization confers a strong cell‐recruiting signal on top of the physical guidance provided by the fiber‐reinforced network. Furthermore, hemolysis assays confirmed the excellent hemocompatibility of the composite. As shown in Figure S14, red blood cells in the FE‐PDA@Fib/Gel‐TG group was completely pelleted with clear supernatants after centrifugation, identical to the saline and Triton X‐100 control groups. Quantitative analysis revealed a hemolysis ratio of less than 5%, well below the permissive safety threshold [46], thus validating its exceptional biosafety for in vivo applications. Taken together, these findings demonstrate that the FE‐PDA@Fib/Gel‐TG composite orchestrates cell behavior through a combination of structural cues (i.e., enhanced stiffness and fiber‐mediated contact guidance) and biochemical cues arising from FE‐mediated chemotaxis, thereby more closely mimicking the regulatory complexity of the bone marrow microenvironment.

During two‐dimensional co‐culture, the initial spatial relationships between MC3T3‐E1 and HUVECs were visualized using lineage‐specific fluorescent probes (green for HUVECs, red for MC3T3‐E1). Immediately after seeding, the two cell populations were largely separated on all substrates (Figure 3h). After 24 h, however, pronounced heterotypic contacts were observed specifically on FE‐PDA@Fib/Gel‐TG, where red and green signals frequently overlapped within localized regions (Figure 3i, blue circles). In contrast, cells on Gel‐TG and PDA@Fib/Gel‐TG remained more dispersed, forming predominantly homotypic clusters with limited direct intertype contact. To further evaluate these interactions, we performed co‐immunofluorescence staining for VE‐cadherin (red, endothelial junction marker) and Runx2 (green, osteogenic transcription factor). As shown in Figure 3j, regions of VE‐cadherin‐positive endothelial structures closely juxtaposed with Runx2‐positive osteogenic cells were most abundant on FE‐PDA@Fib/Gel‐TG. Quantification of overlapping areas (Figure 3k) yielded overlap ratios of 11.9% for Gel‐TG, 11.1% for PDA@Fib/Gel‐TG, and 26.2% for FE‐PDA@Fib/Gel‐TG. Although the absolute number of cells engaged in direct heterotypic proximity remained modest, which is consistent with the known tendency of co‐cultured endothelial and osteogenic cells to preferentially form homotypic aggregates, FE functionalization clearly enhanced the frequency and intimacy of MC3T3‐E1‐HUVEC interactions. Such spatial organization may be relevant to coordinated endothelial‐osteogenic interactions, which are known to contribute to vascularized bone regeneration.

To better recapitulate a three‐dimensional microenvironment, we embedded the co‐cultured cells within the hydrogels to establish a 3D culture system. This configuration allowed cells to interact with the matrix and with each other in all directions, providing a more physiologically relevant environment for network formation. After 3 days, live/dead staining confirmed high cell viability in all groups, and both cell types displayed evident aggregation behavior (Figure 3l). Strikingly, only FE‐PDA@Fib/Gel‐TG supported the formation of extensive, interconnected endothelial‐like network structures, with HUVEC‐derived structures (green) surrounded and partially wrapped by MC3T3‐E1 cells (red), as indicated by the overlapping signals within the circled regions. Quantitative analysis of network architecture (Figure 3m) showed that the number of junctions was increased by approximately 207% and 196% relative to Gel‐TG and PDA@Fib/Gel‐TG, respectively, underscoring the potent pro‐angiogenic effect of FE when presented within the mechanically reinforced composite matrix. Overall, these 2D and 3D in vitro studies demonstrate that FE‐PDA@Fib/Gel‐TG not only maintains excellent biocompatibility but also actively programs endothelial‐osteogenic co‐culture behavior, enhancing cell spreading, migration, heterotypic contact formation, and vascular network assembly.

**FIGURE 4:**
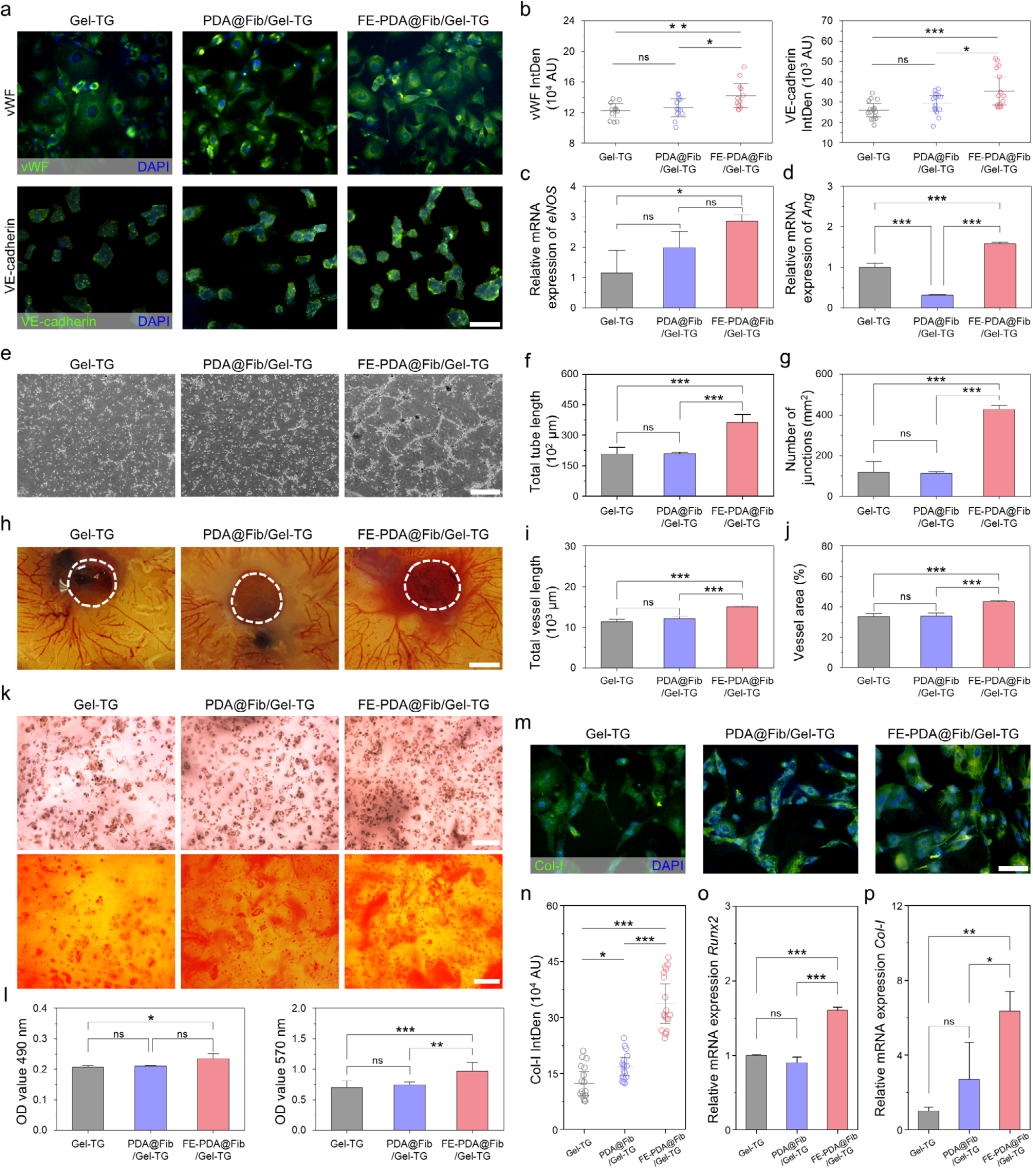
Pro‐angiogenic and osteogenic activities of the engineered hydrogels. (a) Representative immunofluorescence images of endothelial markers vWF and VE‐cadherin in HUVECs cultured on Gel‐TG, PDA@Fib/Gel‐TG and FE‐PDA@Fib/Gel‐TG. (b) Quantification of vWF and VE‐cadherin fluorescence intensity (with a total of 12 randomly selected fields analyzed). (c‐d) Relative mRNA expression of angiogenesis‐related genes *eNOS* and *Ang* in HUVECs on the different hydrogels (*n* = 3). (e) Tube formation assay illustrating in vitro vasculogenic capacity of HUVECs on the various substrates. (f,g) Quantitative analysis of total tube length and number of junctions in (e). (h) Representative images of CAM treated with the different hydrogels; white dashed circles indicate the implantation sites. (i,j) Quantification of total vessel length and vessel area percentage around the implantation sites in (h) (n = 3). (k) ALP staining (upper row) and ARS staining (lower row) of MC3T3‐E1 cells cultured on each hydrogel, indicating early and late osteogenic differentiation. (l) Corresponding quantitative analysis of ALP activity (left) and mineralized matrix deposition (right). (m) Immunofluorescence staining of Col‐I in MC3T3‐E1 cells. (o,p) Relative mRNA expression of osteogenic genes Runx2 and Col‐I in MC3T3‐E1 cells cultured on the different hydrogels. Scale bars: 50 µm in (a), 250 µm in (e), 5 mm in (h), 50 µm (ALP, upper row) and 250 µm (ARS, lower row) in (k). Data are shown as means ± SD. Statistical analysis was performed using one‐way ANOVA with Tukey's post‐hoc test. *p < 0.05, **p < 0.01, and ***p < 0.001; ns, not significant.

The ability of the engineered hydrogels to coordinate angiogenic and osteogenic events was next examined using a series of in vitro cellular experiments. Immunofluorescence staining of HUVECs revealed that scaffold composition strongly influenced the expression of endothelial functional proteins. As shown in Figure 4a, cells on FE‐PDA@Fib/Gel‐TG displayed more intense staining for von Willebrand factor (vWF) and vascular endothelial cadherin (VE‐cadherin) than those on Gel‐TG or PDA@Fib/Gel‐TG. Quantitative analysis confirms the observed visual trend (Figure 4b). The PDA@Fib/Gel‐TG group displays slightly higher expression of vWF and VE‐cadherin compared to the Gel‐TG group. The FE‐PDA@Fib/Gel‐TG composite achieves the highest expression levels among all experimental groups. Specifically, the vWF expression in the FE‐PDA group is approximately 1.4‐fold higher than that in the Gel‐TG group. Furthermore, it is 1.2‐fold higher than the expression in the PDA@Fib/Gel‐TG group. VE‐cadherin levels demonstrate an identical upward trend with similar fold changes. These results indicate that FE presentation on the fiber‐reinforced hydrogel not only preserves endothelial phenotype but also promotes the formation of functional intercellular junctions, which are indispensable for stable vessel formation.

To gain mechanistic insight into the pro‐angiogenic response, we assessed the expression of key angiogenesis‐related genes by qRT‐PCR. During early vascular morphogenesis, endothelial nitric oxide synthase (*eNOS*)‐derived nitric oxide supports HUVEC proliferation and migration, whereas angiopoietin (*Ang*) is critically involved in the maturation and stabilization of nascent sprouts through interactions with mural cells [47]. Consistent with the protein‐level data, HUVECs cultured on FE‐PDA@Fib/Gel‐TG exhibited the highest transcript levels of both genes (Figure 4c,d), with *eNOS* and *Ang* expression increased by 146% and 57%, respectively, relative to Gel‐TG. The functional consequence of these molecular changes was evaluated using a Matrigel tube‐formation assay. As shown in Figure 4e, HUVECs on FE‐PDA@Fib/Gel‐TG formed well‐defined, interconnected capillary‐like networks, whereas cells in the Gel‐TG and PDA@Fib/Gel‐TG groups produced more fragmented and sparsely connected structures, indicative of weaker angiogenic stimulation. Quantitative analysis (Figure 4f,g) revealed that FE‐PDA@Fib/Gel‐TG increased total tube length and junction number by 74.6% and 259.2% over Gel‐TG and by 73.3% and 277.9% over PDA@Fib/Gel‐TG, respectively. To further validate these pro‐angiogenic effects in a more complex biological setting, we employed the chick chorioallantoic membrane (CAM) model. All hydrogels induced some degree of neovascularization around the implantation site, but the FE‐PDA@Fib/Gel‐TG group elicited a markedly denser and more radially organized vascular plexus (Figure 4h). Morphometric analysis confirmed that FE‐PDA@Fib/Gel‐TG increased total vessel length and vessel area by 1.32‐ and 1.28‐fold compared with Gel‐TG, and by 1.24‐ and 1.23‐fold relative to PDA@Fib/Gel‐TG, respectively (Figure 4i,j).

In parallel, we examined the osteogenic performance of the hydrogels using MC3T3‐E1. Early osteogenic differentiation was evaluated by alkaline phosphatase (ALP) staining after 7 d of culture. Cells on FE‐PDA@Fib/Gel‐TG exhibited the most intense and clustered ALP signals compared with those on Gel‐TG or PDA@Fib/Gel‐TG (Figure 4k), indicating robust early osteoblastic activation. After 21 d, Alizarin Red S (ARS) staining revealed extensive mineralized matrix deposition on FE‐PDA@Fib/Gel‐TG, while mineralization on the other substrates was more limited. Quantitative analysis (Figure 4l) showed that ALP activity was highest in the FE group, significantly exceeding that of Gel‐TG and comparable to PDA@Fib/Gel‐TG, whereas the amount of calcium deposition in FE‐PDA@Fib/Gel‐TG was 1.4‐ and 1.3‐fold greater than in Gel‐TG and PDA@Fib/Gel‐TG, respectively. Collagen type I (Col‐I) production, a hallmark of functional osteoblasts and a prerequisite for extracellular matrix mineralization, was further evaluated. Immunofluorescence staining revealed a marked increase in Col‐I signal intensity on FE‐PDA@Fib/Gel‐TG compared to other groups (Figure 4m). Quantification showed that collagen content on FE‐PDA@Fib/Gel‐TG was 174% higher than on Gel‐TG and 101% higher than on PDA@Fib/Gel‐TG (Figure 4n), confirming that FE functionalization greatly enhances matrix synthesis. At the transcriptional level, qRT‐PCR analysis demonstrated significant upregulation of the early osteogenic transcription factor Runx2 and Col‐I in cells cultured on FE‐PDA@Fib/Gel‐TG (Figure 4o,p). Relative to Gel‐TG, Runx2 and Col‐I mRNA levels increased by 1.6‐ and 6.4‐fold, respectively, highlighting a strong genetic commitment toward osteoblastic differentiation. Taken together, these findings show that the FE‐PDA@Fib/Gel‐TG hydrogel not only provides an angiogenic microenvironment that promotes endothelial activation, network formation and vessel growth, but also strongly drives osteogenic differentiation and matrix mineralization. The concurrent enhancement of angiogenic and osteogenic responses suggests that this composite hydrogel is well suited to support tightly coupled vascularized bone regeneration.

**FIGURE 5:**
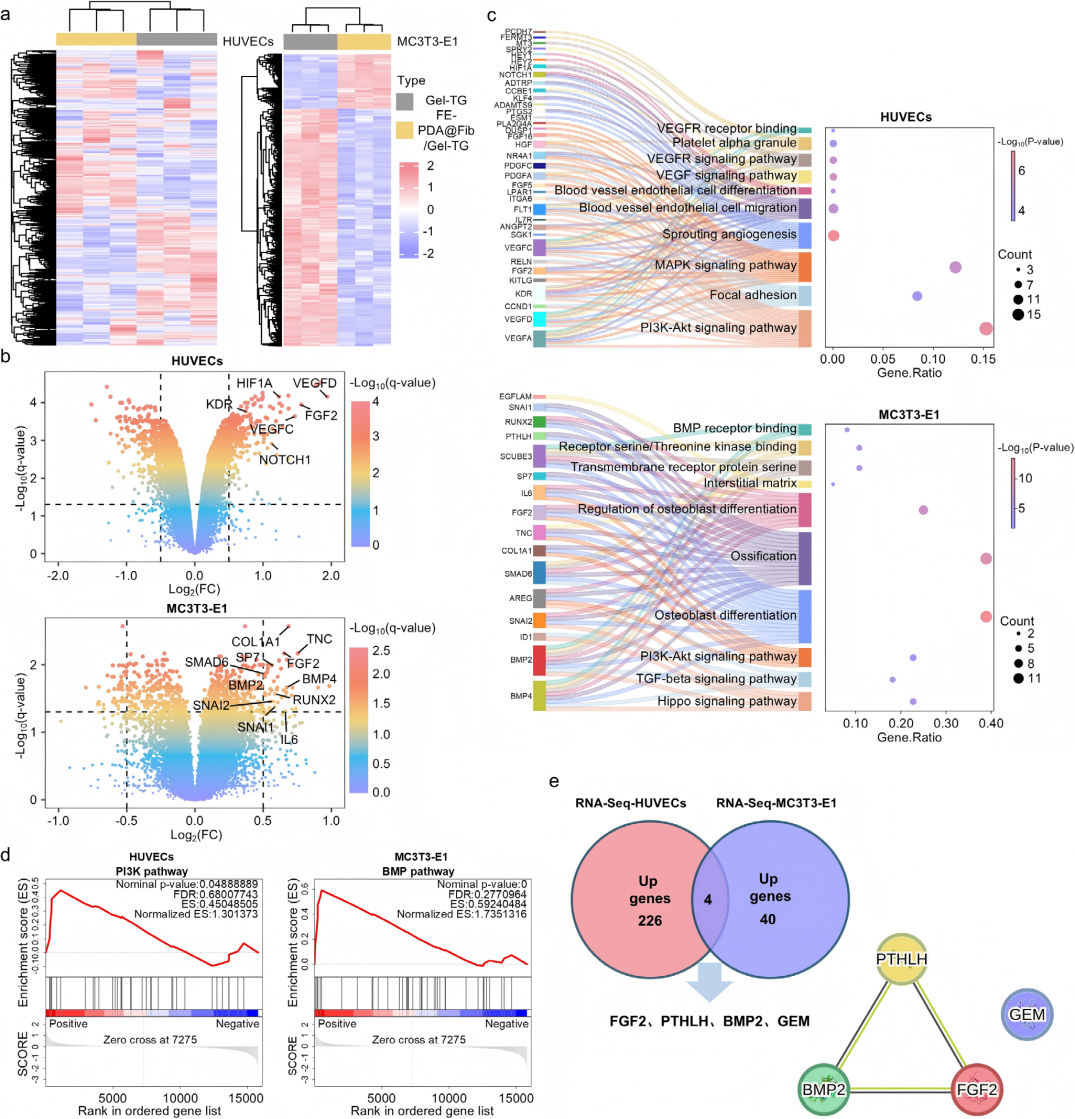
Transcriptomic profiling of HUVECs and MC3T3‐E1 cells cultured on hydrogel substrates. (a) Hierarchical clustering heatmap of gene expression in HUVECs and MC3T3‐E1cells cultured on Gel‐TG or FE‐PDA@Fib/Gel‐TG. (b) Volcano plots displaying differentially expressed genes (DEGs) between FE‐PDA@Fib/Gel‐TG and Gel‐TG. (c) GO term and KEGG pathway enrichment of DEGs in HUVECs (top) and MC3T3‐E1 cells (bottom), shown as Sankey plots linking genes to pathways (left) and corresponding bubble plots summarizing gene ratio and significance (right). (d) Gene set enrichment analysis (GSEA) indicating enrichment of the PI3K signaling pathway in HUVECs and the BMP signaling pathway in MC3T3‐E1 cells cultured on FE‐PDA@Fib/Gel‐TG. (e) Venn diagram of upregulated genes in HUVECs and MC3T3‐E1 cells and associated protein–protein interaction network highlighting shared hub genes *FGF2*, *PTHLH*, *BMP2*, and *GEM*.

To elucidate the molecular basis by which FE‐PDA@Fib/Gel‐TG coordinately regulates angiogenesis and osteogenesis, we performed RNA‐seq on HUVECs and MC3T3‐E1 cells cultured on Gel‐TG or FE‐PDA@Fib/Gel‐TG. Three biological replicates per condition showed high intra‐group consistency, and hierarchical clustering segregated samples according to substrate, indicating robust transcriptional reprogramming induced by the FE‐modified composite (Figure 5a). Differential expression analysis identified 230 upregulated and 278 downregulated genes in HUVECs, and 44 upregulated and 47 downregulated genes in MC3T3‐E1 cells on FE‐PDA@Fib/Gel‐TG compared with Gel‐TG (Figure 5b). Gene Ontology (GO) enrichment analysis revealed that the genes upregulated in HUVECs were predominantly associated with angiogenesis, endothelial cell migration and differentiation, and VEGF/VEGFR signaling. In MC3T3‐E1, enriched GO terms were mainly related to osteoblast differentiation and its regulation. Kyoto Encyclopedia of Genes and Genomes (KEGG) pathway analysis further showed that HUVEC differentially expressed genes (DEGs) were significantly enriched in PI3K‐AKT, focal adhesion, and MAPK signaling, with PI3K‐AKT emerging as the most prominently activated angiogenic axis. In MC3T3‐E1 cells, upregulated genes clustered into Hippo, TGF‐β, and PI3K‐AKT pathways, with a particularly strong enrichment of BMP signaling (Figure 5c,d). Integrating GO and KEGG results, angiogenesis in HUVECs appears to be driven primarily by VEGF/VEGFR‐dependent activation of PI3K‐AKT signaling, with key regulators including *VEGFD*, *VEGFC*, *FGF2*, *HIF1A*, *KDR*, and *NOTCH1*. In MC3T3‐E1 cells, osteogenic commitment is dominated by BMP/Smad and Hippo pathways, with auxiliary input from TGF‐β and MAPK cascades. Canonical osteogenic regulators such as *RUNX2*, *SMAD6*, *SP7*, *COL1A1*, *BMP2* and *BMP4* were among the major contributors (Figure S15a). Of particular interest, FE‐PDA@Fib/Gel‐TG simultaneously upregulated *FGF2*, *PTHLH*, *BMP2*, and *GEM* in both HUVECs and MC3T3‐E1 cells (Figure 5e). FGF2, PTHLH, and BMP2 are well‐known regulators of osteogenesis and bone remodeling, while also functioning as potent pro‐angiogenic mediators, suggesting that these shared factors may act as molecular bridges that couple vascular and bone responses within the composite‐guided microenvironment. Collectively, the transcriptomic data indicate that FE‐PDA@Fib/Gel‐TG enhances angiogenesis in HUVECs mainly through VEGF/VEGFR‐PI3K‐AKT activation, and promotes osteogenesis in MC3T3‐E1 cells via BMP‐ and Hippo‐centered signaling. These findings provide mechanistic evidence that the composite hydrogel orchestrates vascular‐bone coupling at the gene‐regulatory level, thereby underpinning its dual functionality in bone repair (Figure S15b).

### 2.3 In Vivo Bone Regeneration and Angiogenic Potential of Composite Fiber‐Hydrogel

**FIGURE 6:**
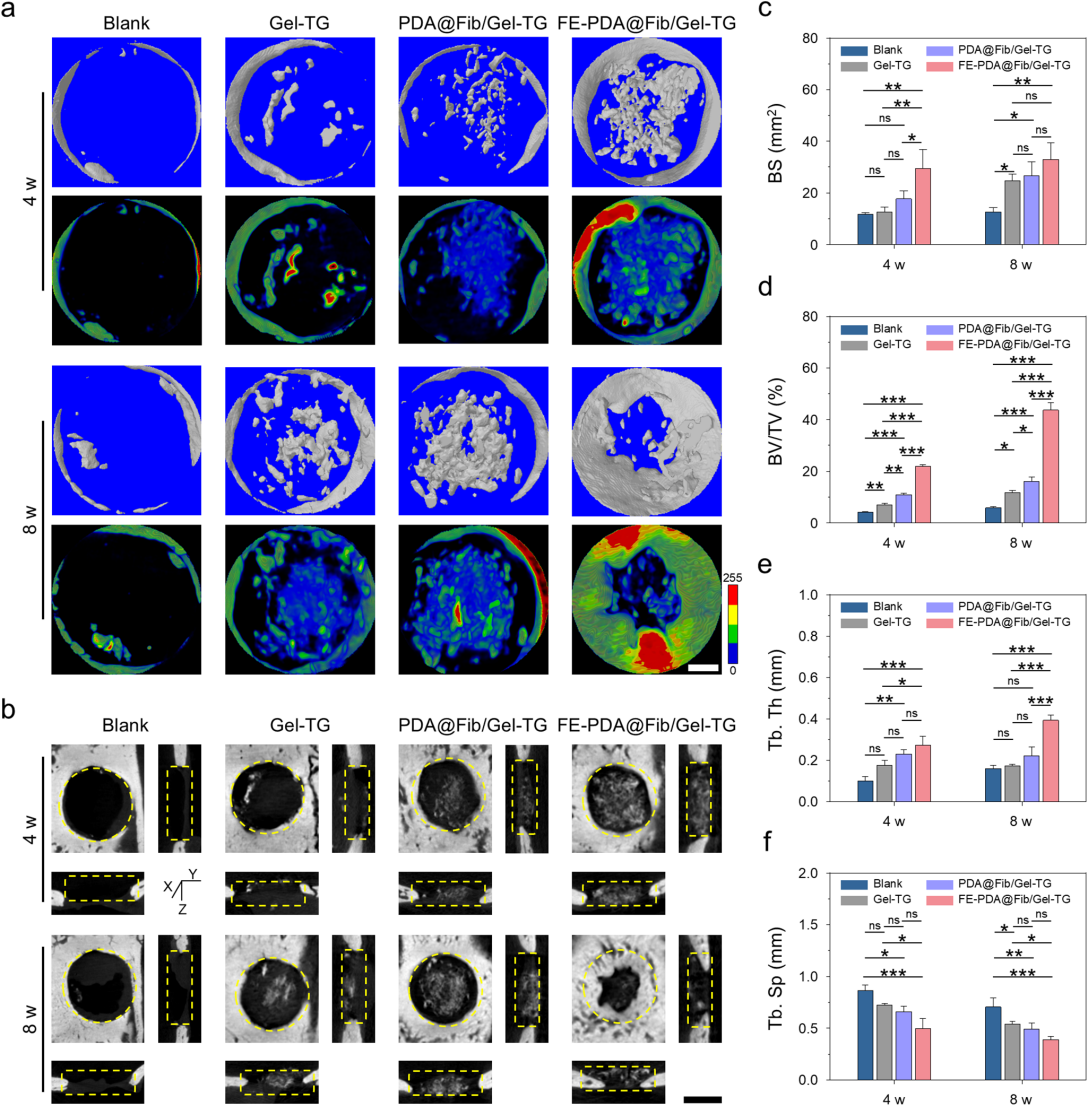
Evaluation of bone regeneration in critical‐sized calvarial defects at 4 and 8 w post‐operation. (a) Representative 3D‐micro‐CT reconstructions and corresponding color‐coded density maps of newly formed bone in Blank, Gel‐TG, PDA@Fib/Gel‐TG and FE‐PDA@Fib/Gel‐TG groups. (b) Representative 2D micro‐CT cross‐sectional images of the defect sites at the same time points. (c–f) Quantitative micro‐CT morphometric analysis of regenerated bone, including bone surface (BS), bone volume fraction (BV/TV), trabecular thickness (Tb.Th) and trabecular separation (Tb.Sp), respectively. Scale bars: 1 mm in (a) and 2.5 mm in (b) (*n* = 5). Data are shown as means ± SD. Statistical analysis was performed using one‐way ANOVA with Tukey's post‐hoc test. *p < 0.05, **p < 0.01, and ***p < 0.001; ns, not significant.

We next examined the capacity of the composite hydrogel to promote bone regeneration and vascular ingrowth in calvarial defect model. Micro‐CT imaging at 4 and 8 weeks captured the evolution of new bone formation within the defects. Three‐dimensional reconstructions showed that in all implanted groups, bone formation initiated from the defect margins and progressed toward the center, whereas almost no mineralized tissue was observed in the Blank defects (Figure 6a). At 4 weeks, the FE‐PDA@Fib/Gel‐TG group already displayed a substantially larger volume of mineralized tissue than the other treatment groups. By 8 weeks, the Blank defects remained largely unbridged, confirming that spontaneous repair is insufficient for this defect size. In contrast, all hydrogel groups supported noticeable bone ingrowth, with FE‐PDA@Fib/Gel‐TG uniquely promoting continuous bone bridging from the periphery toward the center, resulting in near‐complete filling of the defect cavity. Two‐dimensional micro‐CT slices along the X–Y, X–Z, and Z–Y planes provided further structural detail (Figure 6b). At 4 weeks, only sparse new bone was visible in the Blank group, while hydrogels, particularly FE‐PDA@Fib/Gel‐TG, exhibited discernible bony islands extending into the defect. This was most evident in the X–Y view, where FE‐PDA@Fib/Gel‐TG showed contiguous trabecular structures, a pattern that was also apparent in the Z–Y plane. The X–Z sections revealed a clear reduction in defect height, indicating vertical bone apposition. By 8 weeks, defects treated with FE‐PDA@Fib/Gel‐TG showed a markedly reduced central void in all planes, with newly formed bone approaching the thickness of the surrounding native calvaria, whereas residual gaps were still evident in the other groups.

Quantitative micro‐CT analysis of the region of interest corroborated the qualitative observations. Bone surface and bone volume both followed the same trend, with FE‐PDA@Fib/Gel‐TG consistently outperforming the other treatments (Figure 6c, Figure S16a). To more precisely describe the regenerated microarchitecture, we further evaluated bone surface density (BSD) and bone volume fraction (BV/TV) (Figure 6d, Figure S16b). At 4 weeks, FE‐PDA@Fib/Gel‐TG exhibited a BSD of 3.5 mm^−1^, significantly higher than the Blank, Gel‐TG and PDA@Fib/Gel‐TG groups, which showed comparable and much lower values. By 8 weeks, BSD increased in all hydrogel groups and became significantly higher than in the Blank group, yet FE‐PDA@Fib/Gel‐TG still maintained the highest value (4.3 mm^−1^), indicating a more mature and interconnected trabecular network. In line with these findings, BV/TV in the FE‐PDA@Fib/Gel‐TG group reached 21.8% at 4 weeks and 43.7% at 8 weeks, far exceeding the values measured for Blank (4.1% and 5.8%), Gel‐TG (6.9% and 11.6%) and PDA@Fib/Gel‐TG (10.9% and 16.1%). Because trabecular microarchitecture is closely linked to mechanical competence, we further quantified trabecular thickness (Tb.Th), trabecular number (Tb.N) and trabecular separation (Tb.Sp). FE‐PDA@Fib/Gel‐TG yielded significantly higher Tb.Th and Tb.N at both time points compared with all other groups (Figure 6e, Figure S16c). At 4 weeks, Tb.Th and Tb.N in the FE‐PDA@Fib/Gel‐TG group were 2.7 and 2.6 times higher than in Blank, 1.6 and 2.2 times higher than in Gel‐TG, and 1.2 and 1.8 times higher than in PDA@Fib/Gel‐TG. By 8 weeks, the differences became even more pronounced: Tb.Th and Tb.N were 2.5 and 3.3 times higher than in Blank, 2.3 and 1.7 times higher than in Gel‐TG, and 1.8 and 1.4 times higher than in PDA@Fib/Gel‐TG, respectively. Conversely, Tb.Sp was lowest in the FE‐PDA@Fib/Gel‐TG group at both time points (Figure 6f), indicating a dense, well‐connected trabecular network and aligning with the 2D micro‐CT observations. Overall, these in vivo results demonstrate that FE‐PDA@Fib/Gel‐TG markedly accelerates and enhances bone regeneration in critical‐sized defects, yielding higher bone volume, improved trabecular architecture, and more complete defect bridging than either Gel‐TG or PDA@Fib/Gel‐TG alone. Combined with its strong pro‐angiogenic effects, the composite hydrogel provides a conducive microenvironment for coupled vascular and bone tissue ingrowth, thereby supporting effective cranial defect repair.

**FIGURE 7:**
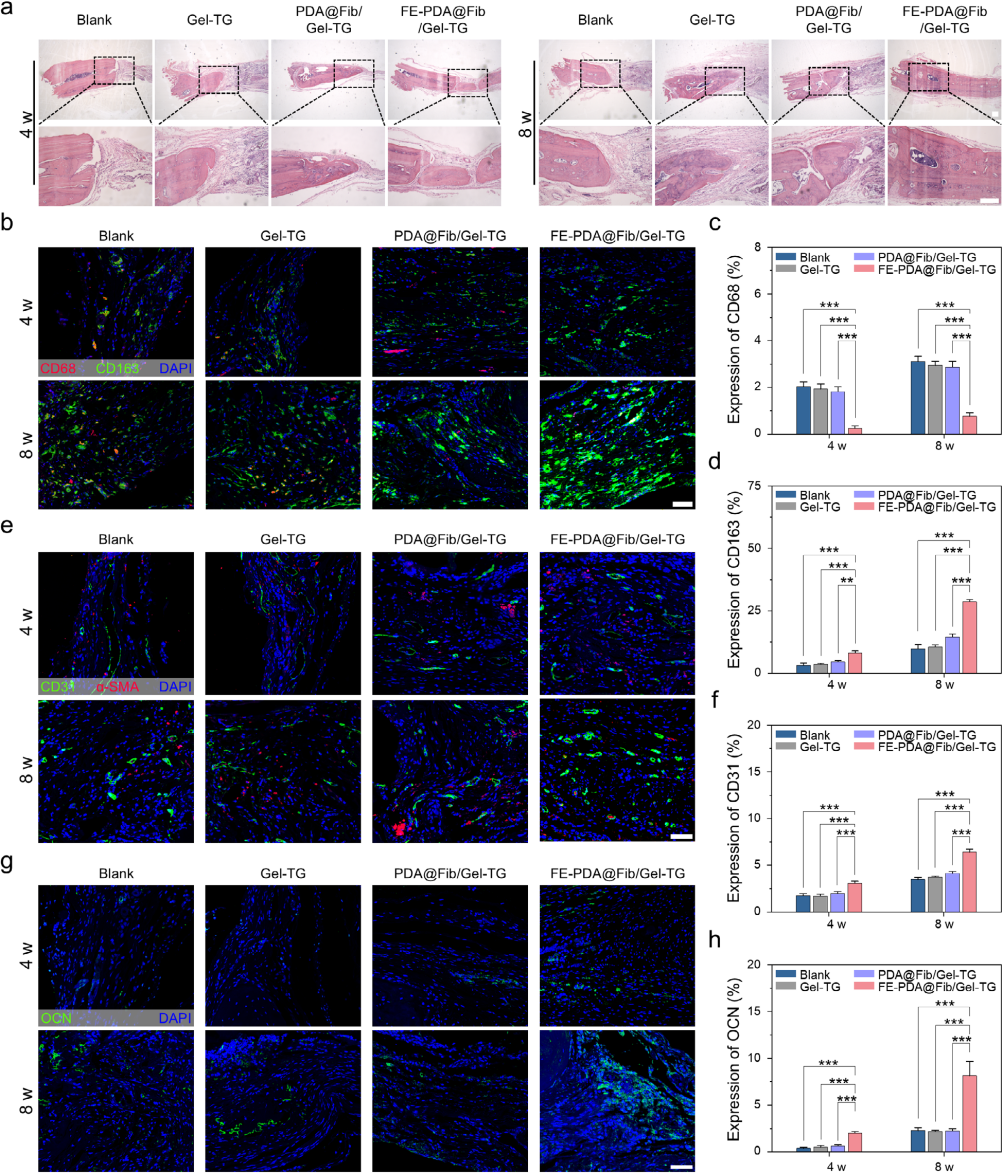
Histological evaluation and immunofluorescence analysis of regenerated tissues at 4 and 8 weeks post‐implantation. (a) H&E staining of calvarial sections showing overall tissue morphology, defect filling, and integration of the different hydrogels with surrounding bone. (b) Immunofluorescence staining of CD68 (red) and CD163 (green) illustrating macrophage infiltration and polarization in the defect regions at each time point. (c‐d) Quantitative analysis of CD68^+^ and CD163^+^ cell fractions, respectively (*n* = 5). (e) Immunofluorescence staining for CD31 (green) and α‐SMA (red) indicating neovascularization and maturation of newly formed vessels. (f) Quantification of CD31 fluorescence intensity (*n* = 5). (g) Immunofluorescence staining for osteocalcin (OCN, green) reflecting osteogenic differentiation within the regenerated tissue. (h) Quantitative analysis of OCN‐positive area (*n* = 5). Scale bars: 200 µm in (a), 50 µm in (b), (e), and (g). Data are shown as means ± SD. Statistical analysis was performed using one‐way ANOVA with Tukey's post‐hoc test. *p < 0.05, **p < 0.01, and ***p < 0.001; ns, not significant.

Histological and immunofluorescence analyses provided further evidence that the hydrogels modulate both bone regeneration and the local immune milieu. H&E staining (Figure 7a) showed no obvious inflammatory infiltrate in any group, indicating good overall biocompatibility. At 4 weeks, the Blank, Gel‐TG and PDA@Fib/Gel‐TG groups were largely filled with loose fibrous tissue and sparsely distributed new bone, whereas defects treated with FE‐PDA@Fib/Gel‐TG contained denser connective tissue, fewer voids, and clear regions of nascent bone matrix at the defect margins. By 8 weeks, all groups displayed increased bone deposition, but the FE‐PDA@Fib/Gel‐TG group exhibited the most extensive and continuous bone formation, with well‐defined osteoid and numerous embedded osteocytes, consistent with markedly enhanced osteogenesis. Masson's trichrome staining (Figure S17) further supported these observations. At 4 weeks, specimens from the Blank, Gel‐TG and PDA@Fib/Gel‐TG groups showed predominantly weak blue staining, indicating limited collagen secretion and immature matrix. In contrast, FE‐PDA@Fib/Gel‐TG sections presented broad blue areas surrounded by emerging red regions, reflecting active collagen deposition and the onset of mineralization. By 8 weeks, the FE‐modified group was dominated by intense red staining, indicative of mature mineralized bone, whereas the other groups still contained large blue regions corresponding to unmineralized tissue.

The immunomodulatory effect of the composite was examined using macrophage markers CD68 (pro‐inflammatory) and CD163 (anti‐inflammatory, M2 phenotype). As shown in Figure 7b, strong CD68 staining was observed in the Blank, Gel‐TG and PDA@Fib/Gel‐TG groups at both time points, while FE‐PDA@Fib/Gel‐TG exhibited notably weaker CD68 signal and a concomitant increase in CD163‐positive cells. Quantitative analysis (Figure 7c) showed that CD68 expression in the FE‐PDA@Fib/Gel‐TG group was reduced by 87.4% and 75.4% relative to Blank at 4 and 8 weeks, and by 86.8% and 74.1% and 85.9% and 73.3% relative to Gel‐TG and PDA@Fib/Gel‐TG, respectively. Conversely, CD163 levels were markedly elevated (Figure 7d), increasing by 113.2% and 155.2% compared with Blank, by 116.5% and 158.4% compared with Gel‐TG, and by 74.5 and 86.7% compared with PDA@Fib/Gel‐TG at 4 and 8 weeks, respectively. These data indicate that FE‐PDA@Fib/Gel‐TG effectively suppresses pro‐inflammatory activation while promoting M2 polarization, thereby establishing an anti‐inflammatory microenvironment that is favorable for tissue repair and bone regeneration. Consistent with the strong pro‐angiogenic capacity observed in vitro, immunostaining for CD31 and α‐SMA revealed more abundant and mature vasculature in the FE‐PDA@Fib/Gel‐TG group (Figure 7e). Quantification of CD31 fluorescence (Figure 7f) showed that endothelial marker expression in this group was 1.73, 1.79, and 1.53 times higher than in Blank, Gel‐TG and PDA@Fib/Gel‐TG at 4 weeks, and 1.84, 1.72, and 1.55 times higher at 8 weeks, respectively, confirming sustained enhancement of neovascularization. Osteocalcin (OCN) staining was used to assess late‐stage osteogenic differentiation. As shown in Figure 7g, OCN fluorescence was weakest in the Blank group and progressively increased with scaffold complexity, with FE‐PDA@Fib/Gel‐TG showing the brightest signal. Quantitative analysis (Figure 7h) revealed that at 4 weeks, OCN levels in the FE‐PDA@Fib/Gel‐TG group (approximately 2.1 arbitrary units) significantly exceeded those in the Blank, Gel‐TG and PDA@Fib/Gel‐TG groups (approximately 0.4, 0.5, and 0.6, respectively). By 8 weeks, OCN expression in FE‐PDA@Fib/Gel‐TG further increased to about 8.2, whereas the other groups remained in a narrow range between 2.2 and 2.3. Taken together, these histological and immunofluorescence data demonstrate that FE‐PDA@Fib/Gel‐TG not only accelerates collagen deposition, mineralization, and bone matrix maturation, but also tunes the immune response toward an M2‐dominant, pro‐healing phenotype while simultaneously promoting angiogenesis. These findings are fully consistent with the in vitro results and further validate the composite hydrogel as a potent immunoregulatory, pro‐angiogenic, and pro‐osteogenic platform for bone regeneration.

## 3 Conclusions

In summary, we engineered a biomimetic composite hydrogel, FE‐PDA@Fib/Gel‐TG, to simultaneously meet the acute and long‐term requirements of cranial defect repair, namely rapid hemostasis and coordinated vascularized bone regeneration. By integrating a chemically crosslinked gelatin network with rigid PDA‐coated HAp/PLLA short fibers and immobilized FE, the construct achieves a favorable combination of mechanics, porosity, degradation kinetics, and controlled release of pro‐regenerative signals. This hierarchically designed microenvironment enhances endothelial‐osteogenic cell adhesion, spreading, migration, and heterotypic crosstalk, activates key angiogenic and osteogenic signaling cascades, and promotes macrophage polarization toward a pro‐healing phenotype, thereby creating a local milieu that is simultaneously hemostatic, pro‐angiogenic, osteoconductive, and immuno‐instructive. Consequently, FE‐PDA@Fib/Gel‐TG drives the formation of dense, well‐organized, and vascularized bone that effectively bridges large cranial defects, with superior trabecular architecture and matrix maturation compared with gelatin or fiber‐reinforced hydrogels alone. The mechanistic insights from transcriptomic analysis further support its ability to couple vascular and bone regeneration through convergent activation of VEGF/VEGFR‐PI3K‐AKT and BMP/Hippo pathways. Taken together, these findings position FE‐PDA@Fib/Gel‐TG as a versatile and potentially translatable platform technology for next‐generation cranial reconstruction. To further elevate its clinical impact, future studies will address current translational limitations by evaluating long‐term in vivo degradation byproducts and optimizing scalability for mass production. Moreover, exploring the precise spatiotemporal dynamics of angiogenesis‐osteogenesis in large animal models will be essential. Ultimately, this work suggests that combining hemostatic control, immune modulation, and spatiotemporally programmed angiogenic‐osteogenic cues within a single injectable scaffold may represent a generalizable strategy for repairing complex osseous defects.

## 4 Experimental Section

### 4.1 Materials

All chemical reagents were used as received without further purification. 2,4,6‐Trinitrobenzenesulfonic acid (TNBS) was purchased from Aladdin (Cat No.: T162601‐1 g). Hydroxyapatite (HAp, 200 nm) was supplied by Macklin. Hexafluoroisopropanol (HFIP, ≥98%) was obtained from Shanghai Darui Fine Chemicals Co. Poly(L‐lactic acid) (PLLA, Mw > 1 000 000) was provided by Jinan Paili Biomaterials Co. Poly(ethylene oxide) (PEO, Mw > 5 000 000) was acquired from Alfa Aesar (UK). Simulated body fluid (SBF) was purchased from MesGen (Cat No.: MG6615). Transglutaminase (1000 U/g) was supplied by Yiming Bio. Gelatin (Type A) and lysozyme were procured from Sigma (USA) and MKBio (Cat No.: MF0202‐25G), respectively. Coumarin 6 (Cat No.: MG35616) was obtained from MesGen Bio. Hydrochloric acid solution (AR grade) and sodium bicarbonate (AR grade) were provided by China National Pharmaceutical Group.

For immunofluorescence staining, the following primary antibodies were used: anti‐vWF (rabbit anti‐rat, Abcam, USA), anti‐VE‐cadherin (rabbit anti‐rat, Abcam, USA), anti‐Collagen I (rabbit anti‐rat, Abcam, USA), anti‐OCN (rabbit anti‐rat, Abcam, USA), anti‐CD68 (rabbit anti‐rat, Abcam, USA), anti‐CD163 (rabbit anti‐rat, Abcam, USA), and anti‐α‐SMA (rabbit anti‐rat, Abcam, USA). All other reagents were of analytical grade unless otherwise specified.

### 4.2 Preparation of Short Fibers

Electrospinning solutions (8% w/v) were prepared by dissolving PLLA, HAp, and PEO in hexafluoroisopropanol (HFIP) at a mass ratio of 7:2:1. High‐molecular‐weight PEO was incorporated to facilitate fiber alignment, while HAp was introduced to mimic the inorganic component of natural bone matrix. Aligned HAp/PLLA fibers were fabricated via stable jet electrospinning under the following parameters: voltage of 4–5 kV, collecting distance of 15 cm, and mandrel (ø = 10 cm) rotation speed of 800 rpm. The resulting fiber mats were vacuum‐dried to remove residual solvent. The dried mats were cut into 3 mm × 3 mm fragments, dispersed in water, and homogenized using a handheld homogenizer to obtain dispersed short fibers. The suspension was passed through a 200‐mesh sieve, and the collected HAp/PLLA short fibers were dehydrated and stored for further use. Furthermore, dopamine hydrochloride was dissolved in 10 mM Tris buffer (pH 8.5) to obtain a 2 mg/mL dopamine solution. The collected short fibers were immersed in the dopamine solution and shaken at 37°C and 300 rpm for 4 h under ambient air to allow spontaneous oxidative polymerization of dopamine. The resulting polydopamine‐coated HAp/PLLA short fibers (PDA@Fib) were rinsed three times with deionized water, frozen at −80°C and lyophilized.

### 4.3 Fiber Morphology Observation

The morphology of fiber mats was examined by SEM. For short fibers, optical microscopy was employed: a small amount of fibers was dispersed in ethanol, and 0.5 mL of the suspension was deposited on a 3.5 mm‐diameter Petri dish. After solvent evaporation, the fibers were imaged, and their lengths were analyzed using ImageJ software. AFM visualizes the mechanical property of fibers.

### 4.4 Hydrogel Preparation

Enzymatically crosslinked gelatin hydrogels were prepared according to a reported method [48]. Briefly, type A gelatin was dissolved in Tris‐HCl buffer at 60°C, and transglutaminase (TGase, 1000 U/g) was separately dissolved in the same buffer. The gelatin and TGase solutions were mixed and pipetted to homogeneity, followed by incubation at room temperature for crosslinking. To optimize crosslinking conditions, hydrogels with three formulations were prepared: 10% gelatin (w/v) with 10 U/mL TGase, 15% gelatin with 15 U/mL TGase, and 20% gelatin with 20 U/mL TGase. The composite hydrogels were prepared through the following procedure: short fibers were first blended with TGase solution, then mixed with gelatin solution, and pipetted to homogeneity. The mixture was crosslinked at room temperature to form composite hydrogels. To optimize fiber content, composites with 0.1%, 0.2%, and 0.5% (w/v) short fibers were prepared.

### 4.5 Preparation of FE‐PDA@Fib/Gel‐TG Hydrogels

The FE used was provided by the Shanghai Ninth People's Hospital (Shanghai, China). Composite hydrogels were prepared in 12‐well plates. Each well was treated with 1.0 mL of 10% FE solution (447.05 ± 12.28 µg/mL) and shaken at 24°C and 300 rpm for 12 h. After removal of unabsorbed FE and rinsing with PBS, the adsorption efficiency was determined using:

(1) $$\mathrm{FE}\;\mathrm{adsorption}\;\mathrm{ratio}\;\;\left(\%\right)=\frac{W_{0}-W_{1}}{W_{0}}\times 100\%$$

where *W_0_* and *W_1_* are the FE contents before and after adsorption, respectively, quantified via BCA assay.

### 4.6 Gelation Time Measurement and Crosslinking Degree Measurement

The gelation time was determined via the vial inversion method. Briefly, 2 mL of the precursor solution was placed in a clear glass vial at room temperature, and the time at which no flow was observed upon inversion was recorded as the gelation time. Images were captured at various time points (30 min, 1 h, 2 h, 4 h, 6 h, 8 h, 10 h, and 12 h) to document structural evolution.

The crosslinking degree of TGase‐mediated gelatin hydrogels was evaluated using a modified TNBS assay. Briefly, 0.05 g of hydrogel was reacted with 1 mL of 0.01% TNBS and 1 mL of 0.1 mol/L sodium bicarbonate. After adding 2 mL of 6 mol/L HCl, the sample was incubated at 60°C for 1.5 h. Absorbance was measured at 355 nm. The crosslinking degree was calculated as:

(2) $$\mathrm{Crosslinking}\;\mathrm{degree}\;\left(\%\right)=\frac{A_{0}-A_{1}}{A_{0}}\times 100\%$$

where *A_0_* and *A_1_* represent the absorbances of non‐crosslinked and crosslinked gelatin, respectively.

### 4.7 Chemical and Structural Characterization

The surface chemical compositions of the composite hydrogels were analyzed via FTIR spectroscopy. To evaluate the crystal structure, lyophilized hydrogel samples were sectioned into 1 mm thick slices. XRD analysis was performed over a 2θ range of 5° to 80° at a scan rate of 5°/min under 40 kV and 300 mA.

### 4.8 Mechanical and Rheological Evaluation

Compressive mechanical testing: Cylindrical hydrogel samples (ø 10 mm × 10 mm height) prepared in a 48‐well plate were subjected to uniaxial compression tests at a displacement rate of 5 mm/min until failure. Dynamic frequency sweep tests: A rheometer performs the evaluation using a 20 mm parallel plate. The sample gap measures 1 mm. The testing temperature remains at 25°C. The instrument applies a fixed 1% oscillatory strain. The angular frequency sweeps from 0.1 to 100 rad/s.

### 4.9 Swelling, Degradation, and Bioactivity

Water absorption ratio: The weight of dry hydrogel (*W_1_*) was recorded before immersion in PBS at 37°C for 12 h. The swollen hydrogel was gently blotted and weighed (*W_2_*). The water absorption ratio was calculated as:

(3) $$\mathrm{Water}\;\mathrm{absorption}\;\mathrm{ratio}\;\;\left(\%\right)=\frac{W_{2}-W_{1}}{W_{1}}\times 100\%$$

Degradation profile: Hydrogel degradation was evaluated in lysozyme‐containing PBS (10^5^ U/mL, pH 7.2) at 37°C with shaking at 90 rpm. Samples were retrieved at designated time points, rinsed, dried, *M_0_* is the initial weigh and weighed (*M_t_*). The degradation ratio was calculated as:

(4) $$\mathrm{Degradation}\;\mathrm{ratio}\;\left(\%\right)=\frac{M_{0}-M_{t}}{M_{0}}\times 100\%$$

Mineralization assay: Hydrogel samples (ø 10 mm × 10 mm) were incubated in 8× simulated body fluid at 37°C with shaking for 3 days. After rinsing and lyophilization, apatite deposition was examined using SEM.

### 4.10 FE Release Profile

FE‐loaded hydrogels (Gel‐TG and PDA@Fib/Gel‐TG) were immersed in PBS at 37°C under agitation. Supernatant (20 µL) was collected and replenished with fresh PBS at predetermined intervals (1–20 h). FE concentration was quantified via BCA assay.

### 4.11 Cell Culture and In Vitro Biocompatibility

HUVECs and MC3T3‐E1 cells were co‐cultured at a 1:1 ratio in mixed endothelial/osteogenic medium and seeded onto hydrogels. Gelatin was autoclaved, enzyme solutions were filter‐sterilized, and short fibers were UV‐ and ethanol‐treated. Hydrogel precursors (500 µL/well) were crosslinked for 12 h in 12‐ or 24‐well plates. Furthermore cells were cultured on hydrogels for 1–5 days, incubated with MTT solution for 4 h, followed by DMSO addition. Absorbance was measured at 490 nm.

### 4.12 Cell Staining and Imaging

After 24 h of culture, fluorescence staining was used to assess cell morphology. For SEM, cells were fixed, dehydrated in an ethanol series, lyophilized, and sputter‐coated. Images were false‐colored using Adobe Photoshop to enhance cell visibility. Viability was evaluated via live/dead staining.

### 4.13 In Vitro Hemolysis Assay

Fresh rat blood was collected and centrifuged (1000 rpm, 10 min) to harvest red blood cells (RBCs). The RBCs were diluted with normal saline and incubated with the FE‐PDA@Fib/Gel‐TG hydrogels at 37°C for 2 h. RBC suspensions treated with Triton X‐100 and normal saline served as the positive and negative controls, respectively. After incubation, the mixtures were centrifuged (1200 rpm, 10 min), and the supernatants were collected. A microplate reader was used to record the absorbance of the supernatants at 540 nm, from which the hemolysis ratio was calculated.

### 4.14 Cell‐Cell Interactions and Functional Evaluation

To investigate the synergistic effects of the co‐culture system, the following functional assays were performed:

Transwell invasion: Cell invasion was assessed using Transwell inserts placed over hydrogels. Migrated cells were quantified after 24 h. Cell‐cell interaction: In 2D, pre‐labeled HUVECs and MC3T3‐E1 were co‐cultured, fixed, and imaged; immunofluorescence was performed using anti‐VE‐cadherin and anti‐Runx2 antibodies. Overlap rates were quantified with ImageJ. For 3D tube formation, pre‐stained cells were embedded in Matrigel–hydrogel mixtures, cultured for 72 h, and network formation was imaged and quantified.

### 4.15 Endothelial Gene and Protein Expression

Co‐cultured cells (1:1, 2.0 × 10^4^ cells/well) were seeded on hydrogels for 3 days. Immunofluorescence staining for vWF and VE‐cadherin was performed according to the manufacturer's protocols. The expression of angiogenesis‐related genes (*eNOS* and *Ang*) was analyzed via qRT‐PCR using the primers listed in Table S1.

### 4.16 Evaluation of Angiogenic Potential

Tube formation assay: A tube formation assay was conducted in 35 mm confocal dishes with a 15 mm borosilicate glass bottom. Hydrogels (Gel‐TG, PDA@Fib/Gel‐TG, FE‐PDA@Fib/Gel‐TG) were formed in the outer wall region. Then, 200 µL of Matrigel was evenly applied to the glass lumen area and solidified at 37°C for 0.5 h. Co‐cultured cells (1:1, 3.0 × 10^4^ cells/well) were seeded onto the Matrigel and allowed to adhere for 1 h at 37°C with 5% CO_2_. After 18 h of culture, endothelial network structures were imaged and quantified for tube length and junctions using ImageJ. Chorioallantoic membrane assay: Hydrogel samples (Gel‐TG, PDA@Fib/Gel‐TG, FE‐PDA@Fib/Gel‐TG) with a diameter of 10 mm and height of 2 mm were prepared and evaluated using the chick chorioallantoic membrane model.

### 4.17 Osteogenic Differentiation and Mineralization

ALP staining and quantification: Co‐cultured HUVECs and MC3T3‐E1 cells (1:1 ratio, 2.0 × 10^4^ cells/well) were seeded on various hydrogels (Gel‐TG, PDA@Fib/Gel‐TG, FE‐PDA@Fib/Gel‐TG). The medium was changed every two days. After 7 days, ALP staining and quantification were performed using a commercial kit according to the manufacturer's instructions. Alizarin Red S (ARS) staining and quantification: Cells were cultured under the same conditions for 21 days. ARS staining and quantification were then conducted to assess mineral deposition.

### 4.18 Osteogenic Gene and Protein Expression

The expression of osteogenic genes (*Runx2* and *Col‐I*) was evaluated via qRT‐PCR using the primers listed in Table S2.

For protein expression analysis, co‐cultured cells were fixed on day 5 and immunostained for Col‐I.

### 4.19 Transcriptome Sequencing and Bioinformatics Analysis

Total RNA was extracted from HUVECs and MC3T3‐E1 treated with Gel‐TG or FE‐PDA@Fib/Gel‐TG (*n* = 3). Transcriptome sequencing was performed on the Illumina HiSeq platform to generate gene expression matrices. Differential expression analysis was conducted with the limma R package (version 3.52.4) [49], using thresholds of *p* < 0.05 and |log_2_FC| > 0.5. Gene expression data were visualized with ggplot2 (version 3.5.1) [50]. KEGG and GO enrichment analyses were performed using ClusterProfiler (version 4.12.6) and GSVA (version 1.52.3) [51]. GSEA was conducted with GSEABase (version 1.66.0). Protein–protein interaction networks were constructed using the STRING database (https://cn.string‐db.org/). All analyses were conducted in the R environment (version 4.4.3; https://www.r‐project.org/).

### 4.20 In Vivo Skull Defect Repair

A critical‐sized calvarial defect model (5 mm diameter) was established in 32 male Sprague‐Dawley rats (∼400 g). Defects were created bilaterally using a trephine drill. Hydrogel implants (5 mm diameter, 2 mm height) were assigned to four groups. Implants were placed randomly into the defects, and wounds were sutured. Animals were euthanized at 4 and 8 w post‐surgery for imaging and histological analysis. All procedures were approved by the Animal Care and Use Committee of Shanghai Jiao Tong University School of Medicine (Approval No. JUMC2025‐301‐B).

### 4.21 Radiographic and Histological Evaluation

Micro‐CT analysis: Harvested calvaria were fixed in 4% PFA for 3 days and scanned using a micro‐CT system under the following parameters: voltage 65 kV, current 380 µA, resolution 18 µm, filter 1.0 mm. SkyScan Data Viewer and CT‐Analyzer software were used for 3D reconstruction and morphometric analysis, including bone surface (BS), bone volume (BV), bone surface density (BSD), bone volume fraction (BV/TV), trabecular thickness (Tb.Th), and trabecular number (Tb.N). H&E staining: Samples were decalcified in 10% EDTA for approximately 21 days, dehydrated, and embedded in paraffin. Sections (5 µm thickness) were stained with H&E using standard procedures and imaged under a light microscope. Immunofluorescence staining: Immunofluorescence was performed to detect immune markers (CD68, CD163), vascular markers (CD31, α‐SMA), and OCN. Sections were blocked with 10% goat serum, incubated with primary antibodies overnight, and visualized using a fluorescence microscope. Quantification was performed with ImageJ.

### 4.22 In Vivo Hemostatic Evaluation

We assessed the hemostatic performance of the materials in a rat tail transection model. Briefly, male Sprague‐Dawley rats (≈400 g) were anesthetized with 1% pentobarbital. We then amputated 60% of the tail length after positioning a pre‐weighed filter paper beneath it. Following 5 s of free bleeding, we applied the PDA@Fib/Gel‐TG hydrogel to the wound in the experimental group. We recorded the hemostasis time and quantified the blood loss and the Gel‐TG and blank groups served as controls (n = 3). The blood loss was calculated according to:

(5) $$Bloodloss\left(g\right)=\left(M_{F}-M_{f}\right)-\left(M_{D}-M_{f}\right)$$

Here, *M_d_* and *M_f_* are the initial dry weights of the filter paper and material, while *M_F_* and *M_D_* are their final wet weights post‐experiment.

### 4.23 Statistical Analysis

Statistical analysis in this study was performed using embedded algorithms in the commercial software GraphPad Prism 8. All quantitative analyses were performed using Image J software (V.1.52a). All results are represented as mean ± standard deviation from at least three independent experiments. All analyses were conducted using one or two‐way analysis of variance (ANOVA) with Tukey's post‐hoc test. A value of *p*< 0.05 was considered statistically significant, and ns represents no significance (∗*p*< 0.05, ∗∗*p*< 0.01 and ∗∗∗*p*< 0.001).

## Author Contributions

Conceptualization: D. Sha, S. Wang, and D. Song. Methodology: L. Che, D. Li, and H. Zhang. Experiments and characterizations: L. Che, D. Sha, D. Li, T. Xu, and H. Zhang. post – editing: D. Sha, D. Li, and H. Zhang. Schematic diagrams: D. Sha, L. Che, D. Li, and J. Li. Data processing in the main text: L. Che, D. Li, X. Chen, and D. Sha. Data processing in the supporting text: J. Li. Funding acquisition: L. Che. Project administration: L. Che, D. Sha, S. Wang, and D. Song. Supervision: D. Sha, S. Wang, L. and D. Song. Writing – original draft: D. Sha, L. Che, D. Li. Writing – review & editing: D. Li, L. Che, D. Sha, S. Wang, L. D. Zhang, and D. Song.

## Funding

This work was supported by the National Natural Science Foundation of China (No. 82202675) and the Preliminary Research Fund of Shanghai General Hospital (No. YY202510).

## Ethics Statement

The experimental protocol was approved by the Animal Care and Use Committee of Shanghai Jiao Tong University School of Medicine (Approval No. JUMC2025‐301‐B).

## Conflicts of Interest

The authors declare no conflicts of interest.

## Supporting information




**Supporting File**: advs75590‐sup‐0001‐SuppMat.pdf.

## Data Availability

The data that support the findings of this study are available from the corresponding author upon reasonable request.
