# Supplementary material for: A Bone Marrow–Mimetic Hydrogel Enables Dual‐Phase Hemostasis and Vascularized Osteogenesis for Cranial Defects
Source: Adv Sci (Weinh). 2026 May 8;13(43):e75590. doi: 10.1002/advs.75590 (PMC13335774; doi:10.1002/advs.75590)
Supplement: Supplementary file 1 — Supporting File: advs75590‐sup‐0001‐SuppMat.pdf. [file ADVS-13-e75590-s001.pdf]

Supporting Information

A Bone Marrow–Mimetic Hydrogel Enables Dual-Phase Hemostasis and Vascularized Osteogenesis for Cranial Defects

Lingbin Che[+], Donghong Li[+], Huan Zhang[+], Tingting Xu, Juhan Li, Xuanzhou Chen, Louis D. Zhang, Shuguang Wang[*], Dianwen Song[*] and Dongyong Sha[*]

L. Che, J. Li, D. Song, D. Sha.
Department of Orthopedics, Shanghai General Hospital, Shanghai Jiao Tong University School of Medicine, Shanghai 200080, China.
E-mail: (shadongyong@sjtu.edu.cn, D. Sha; dwsong@sjtu.edu.cn, D. Song)
D. Li, T. Xu.
College of Biological Science and Medical Engineering Donghua University Shanghai 201620, China.
H. Zhang.
Department of Emergency Medicine, The Affiliated Hospital of Xuzhou Medical University, School of Second Clinical Medicine of Xuzhou Medical University, Xuzhou, Jiangsu, 221002, China.
X. Chen
School of Electrical and Computer Engineering, Georgia Institute of Technology, Atlanta, Georgia 30332, USA.
D. Zhang.
Power Dream America, Inc., Peachtree Corners, GA 30071, USA.
S. Wang.
Trauma Center, Shanghai General Hospital Shanghai Jiao Tong University School of Medicine, Shanghai 200080, China.
E-mail: (shuguangw@sjtu.edu.cn, S. Wang)
+ L. Che, D. Li, and H. Zhang contributed equally to this work.

This file includes the following:

Tables S1-S2

Figures S1-S17

Table S1. Primer sequences for angiogenesis-related genes.

**Gene**	**Forward primer (5′–3′)**	**Reverse primer (5′–3′)**
Ang	CTGGGCGTTTTGTTGTTGGTC	GGTTTGGCATCATAGTGCTGG
eNOS	CCGAGTCCTCACCGCCTTCT	GGTAACATCGCCGCAGACAAA
GAPDH	GGACCTGACCTGCCGTCTAG	GTAGCCCAGGATGCCCTTGA

Table S2. Primer sequences for osteogenesis-related genes.

Gene	Forward primer (5′–3′)	Reverse primer (5′–3′)
Col-I	TTCCTGAAGATGTCGTTGATGTG	TGTTTTTGCAGTGGTATGTGTAATGTTC
Runx2	GACTGTGGTTACCGTCATGGC	ACTTGGTTTTTCATAACAGCGGA
GAPDH	CAAGCTCATTTCCTGGTATGACAAT	GTTGGGATAGGGCCTCTCTTG

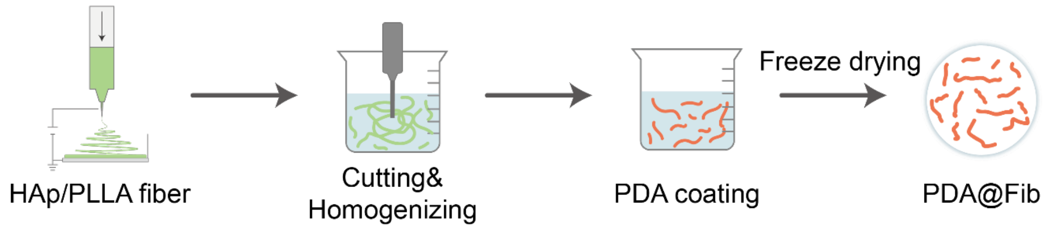

Figure S1. Schematic illustration of the fabrication of PDA@Fib, showing electrospinning of HAp/PLLA fibers, fragmentation into short fibers, and subsequent polydopamine surface coating.

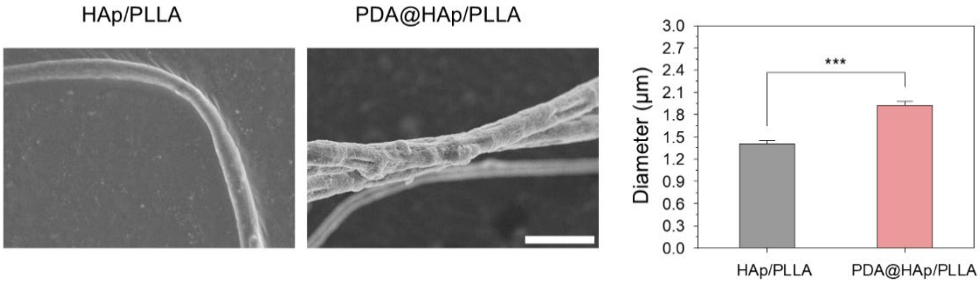

Figure S2. Morphological characterization and diameter quantification of the composite fibers. Scale bar: 5 μm.

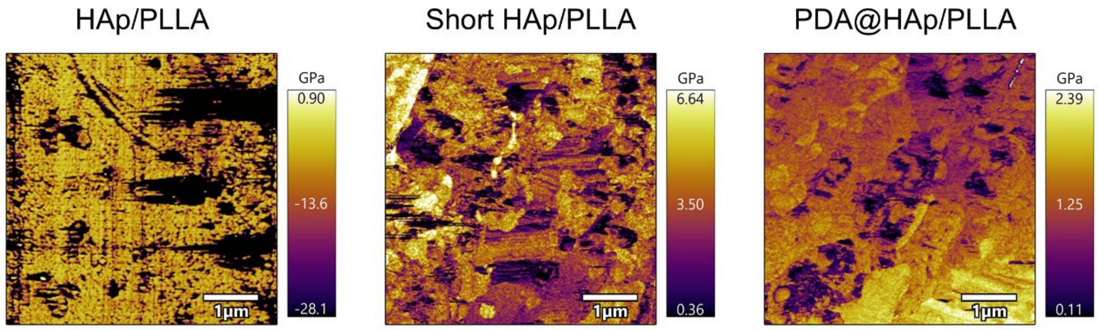

Figures S3. Atomic force microscopy modulus maps of different fibers.

.

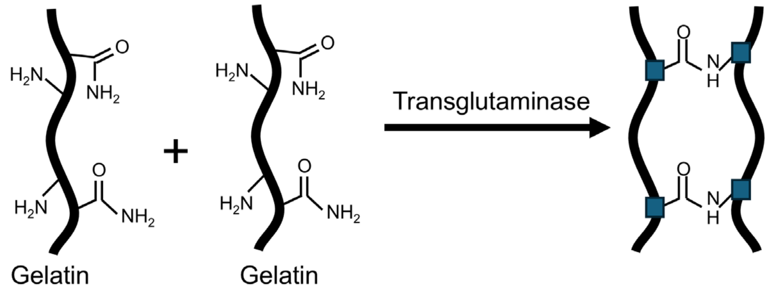

Figure S4. Schematic illustration of TGase-catalyzed crosslinking of gelatin chains *via* formation of ε-(γ-glutamyl)lysine bonds between glutamine and lysine residues.

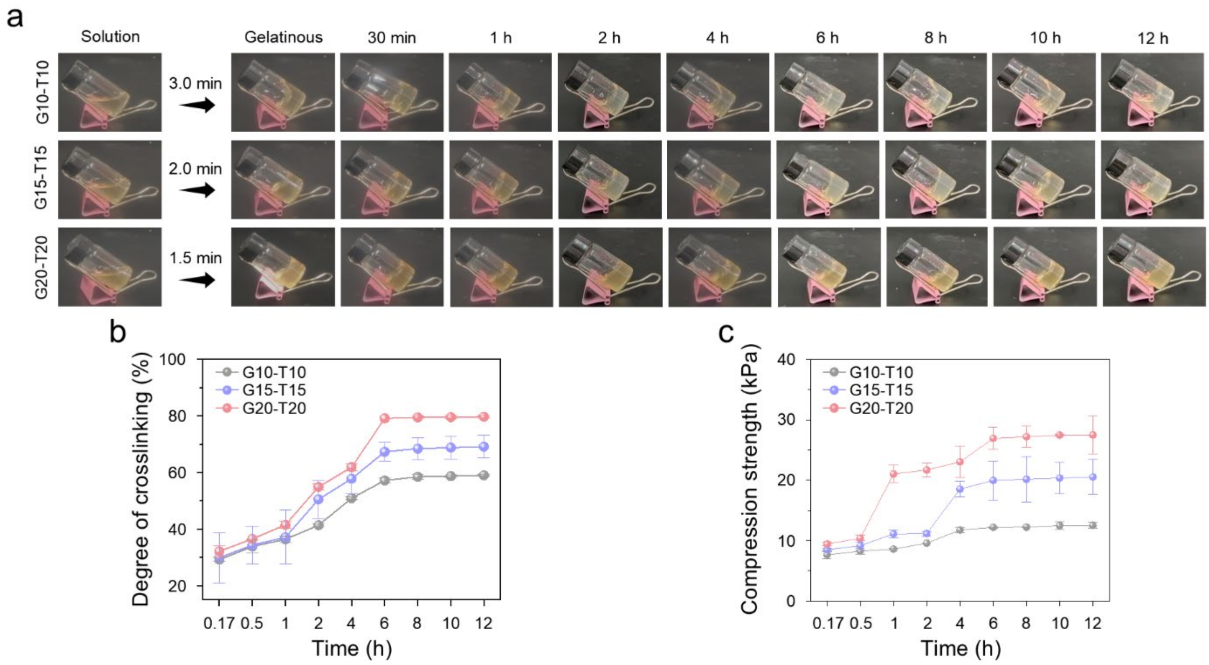

Figure S5. Preparation and characterization of Gel-TG hydrogels. (a) Photos of gelling hydrogels taken at different time points; (b) Determination of cross-linking degree; (c) Compressive strength of hydrogels at different time points. (Note: Due to the low strength of the hydrogel gelled for a short period of time (10 min), it broke after applying a compressive strain of >10%, therefore the compressive strength determined are based on the results of the hydrogels compressed at 10% of strain).

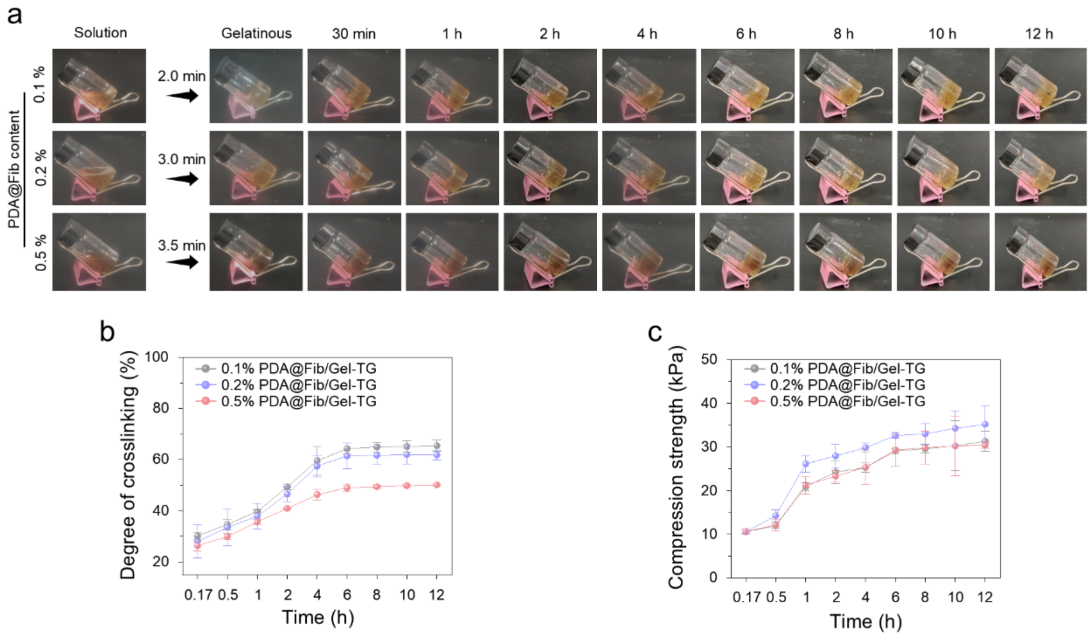

Figure S6. Preparation and characterization of PDA@Fib/Gel-TG composite hydrogels. (a) Photos of the gelling composite hydrogels taken at different time points; (b) Determination of cross-linking degree; (c) Compressive strength of the composite hydrogels at different time points. (Note: Due to the low strength of the hydrogel gelled for a short period of time (10 min), it broke after applying a compressive strain of >10%, therefore the compressive strength determined are based on the results of the composite hydrogels at 10% of strain).

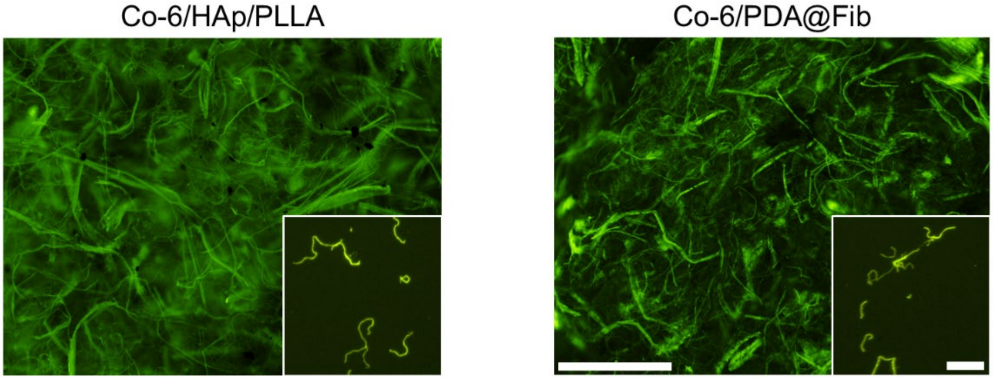

Figure S7. Fluorescent images of the short fibers doped by coumarin 6 (Co-6) before and after PDA coating. Scale bars: 100 µm (main image) and 10 µm (inset).

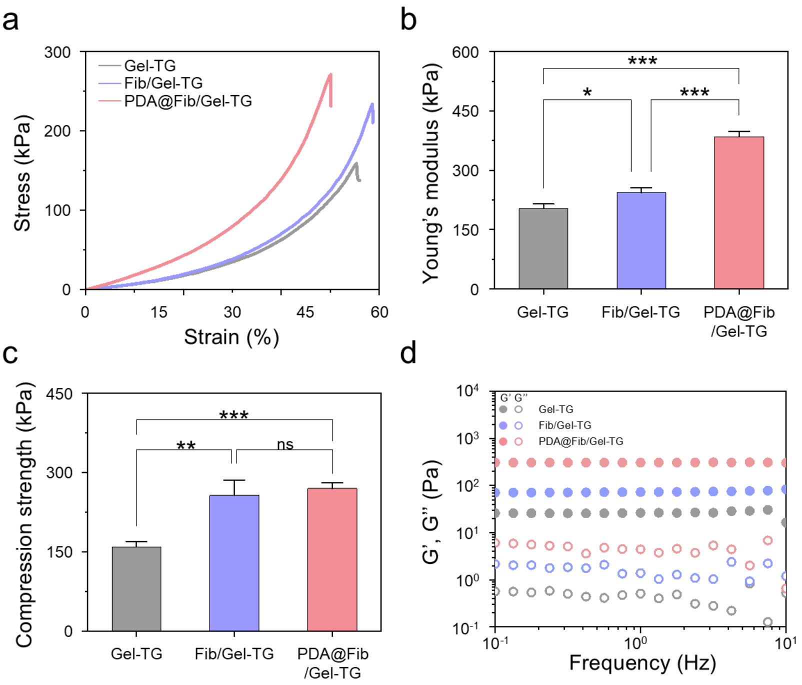

Figure S8. Mechanical performance of the hydrogels under uniaxial compression. (a) Compressive stress-strain curves and (b) corresponding Young's modulus of Gel-TG, Fib/Gel-TG, and PDA@Fib/Gel-TG hydrogels (n=3). (c) Compressive strength of composite hydrogels (n=3). (d) Frequency-dependent rheological behavior of gels. Data are shown as means ± SD. Statistical analysis was performed using one-way ANOVA with Tukey's post-hoc test. *P < 0.05, **P < 0.01, and ***P < 0.001; ns, not significant.

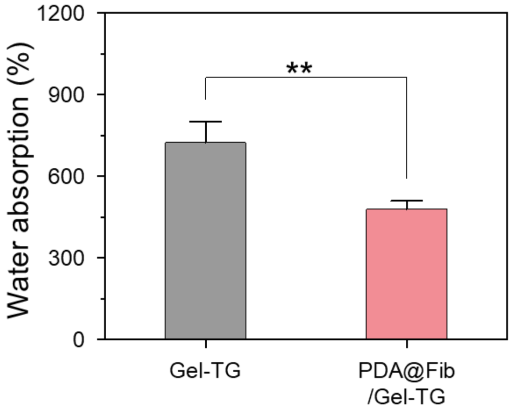

Figure S9. Water absorption of hydrogels (n=3). Data are shown as means ± SD. Statistical analysis was performed using one-way ANOVA with Tukey's post-hoc test. *P < 0.05, **P < 0.01, and ***P < 0.001; ns, not significant.

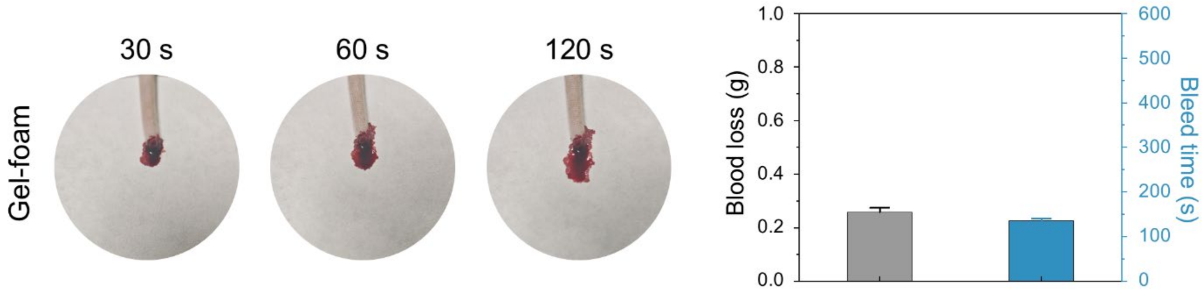

Figure S10. *In vivo* hemostatic performance in a mouse tail hemorrhage model. The images display representative photographs of bleeding mouse tails treated with Gel-foam at different time points. The bar charts show the corresponding quantitative analyses of total blood loss volume and hemostasis time.

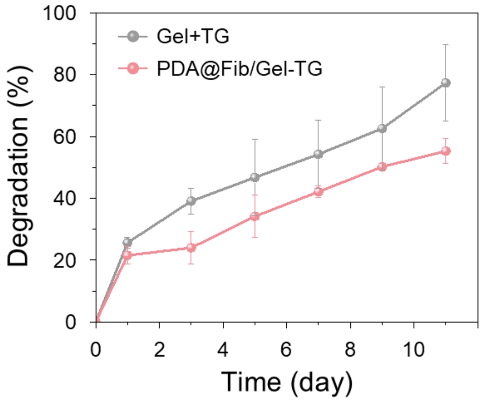

Figure S11. Enzymatic degradation profiles of Gel-TG and PDA@Fib/Gel-TG hydrogels.

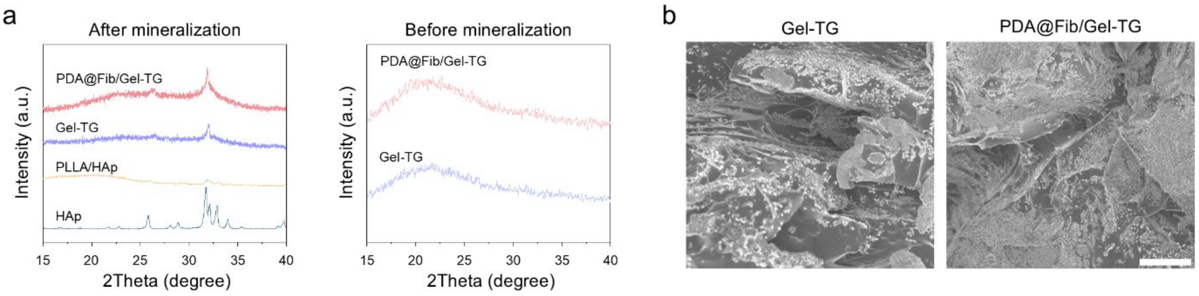

Figure S12 Morphology and crystalline composition of mineralized hydrogels (a) XRD diffraction patterns of HAp, HAp/PLLA fibers, and the composite hydrogels before and after mineralization. (b) SEM images showing mineral deposits formed on Gel-TG (left) and PDA@Fib/Gel-TG (right) after 3 days of mineralization. Scale bar: 50 µm.

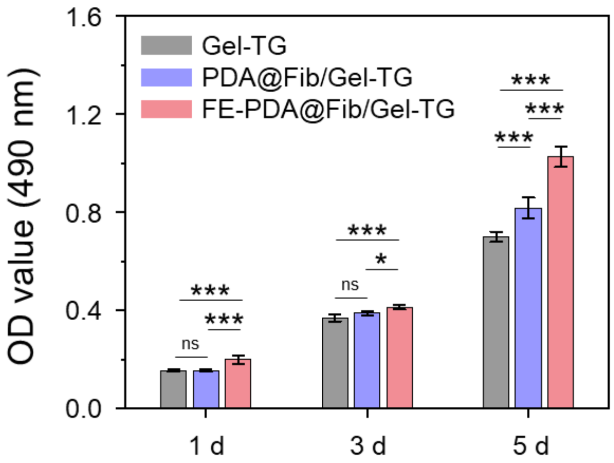

Figure S13. Cell proliferation on the different hydrogels quantified by MTT assay at predetermined time points (n=3). Data are shown as means ± SD. Statistical analysis was performed using one-way ANOVA with Tukey's post-hoc test. *P < 0.05, **P < 0.01, and ***P < 0.001; ns, not significant.

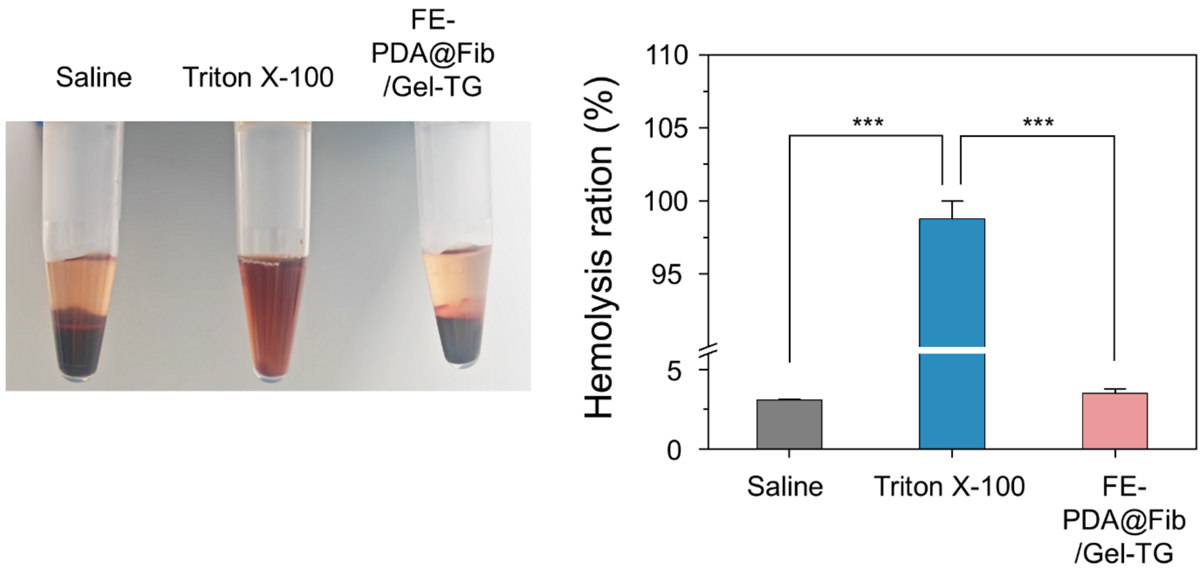

Figure S14. Macroscopic observation and quantitative analysis of the hemolysis ratio for red blood cells incubated with saline (negative control), Triton X-100 (positive control), and the FE-PDA@Fib/Gel-TG hydrogel (n=3). Data are shown as means ± SD. Statistical analysis was performed using one-way ANOVA with Tukey's post-hoc test. *P < 0.05, **P < 0.01, and ***P < 0.001; ns, not significant.

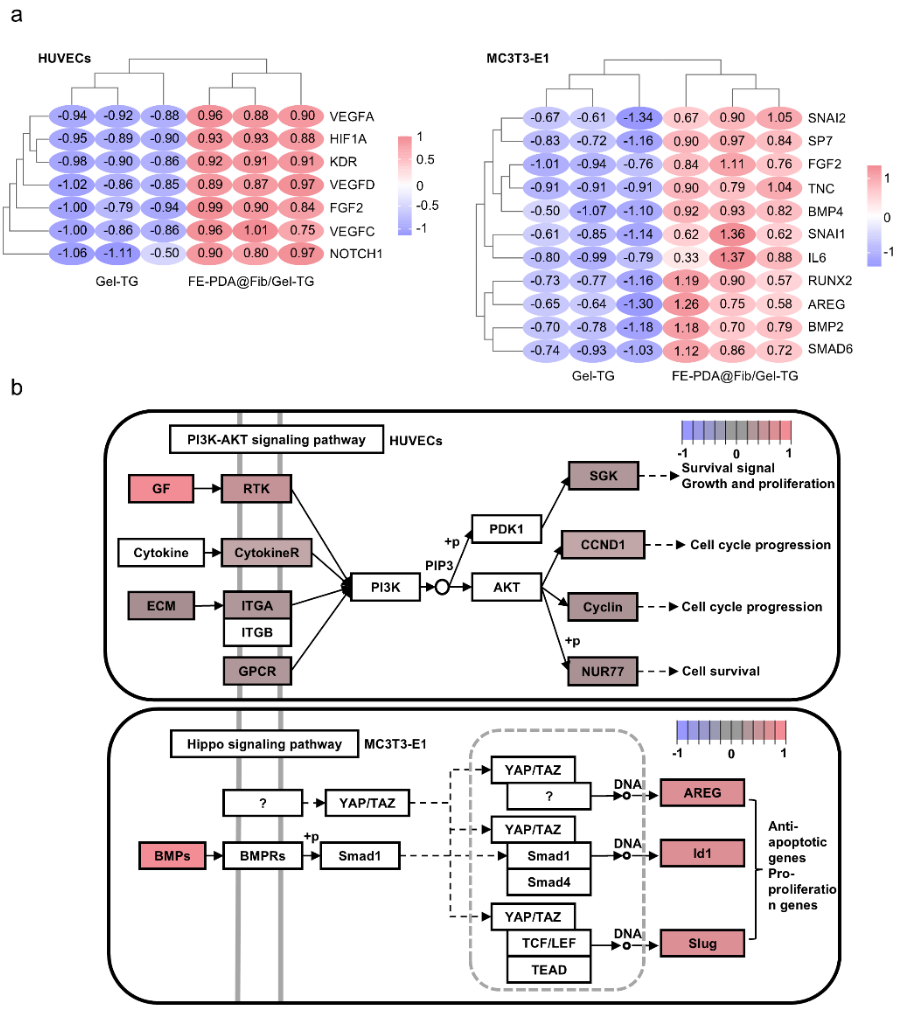

Figure S15. Molecular mechanisms underlying the pro-angiogenic and osteogenic effects of FE-PDA@Fib/Gel-TG hydrogel. (a) Key angiogenesis-related genes upregulated in HUVECs and key osteogenesis-related genes upregulated in MC3T3-E1 following FE-PDA@Fib/Gel-TG treatment. (b) Schematic diagrams illustrating the PI3K-AKT pathway in HUVECs and the Hippo pathway in MC3T3-E1.

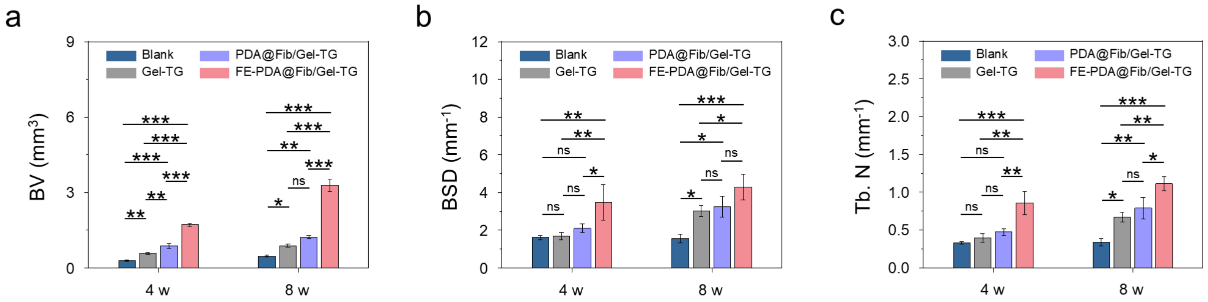

Figure S16. Determination of bone tissue morphometric parameters. (a) Bone volume (BV). (b) Bone surface density (BSD). (c) Number of trabeculae (Tb.N) (n=5). Data are shown as means ± SD. Statistical analysis was performed using one-way ANOVA with Tukey's post-hoc test. *P < 0.05, **P < 0.01, and ***P < 0.001; ns, not significant.

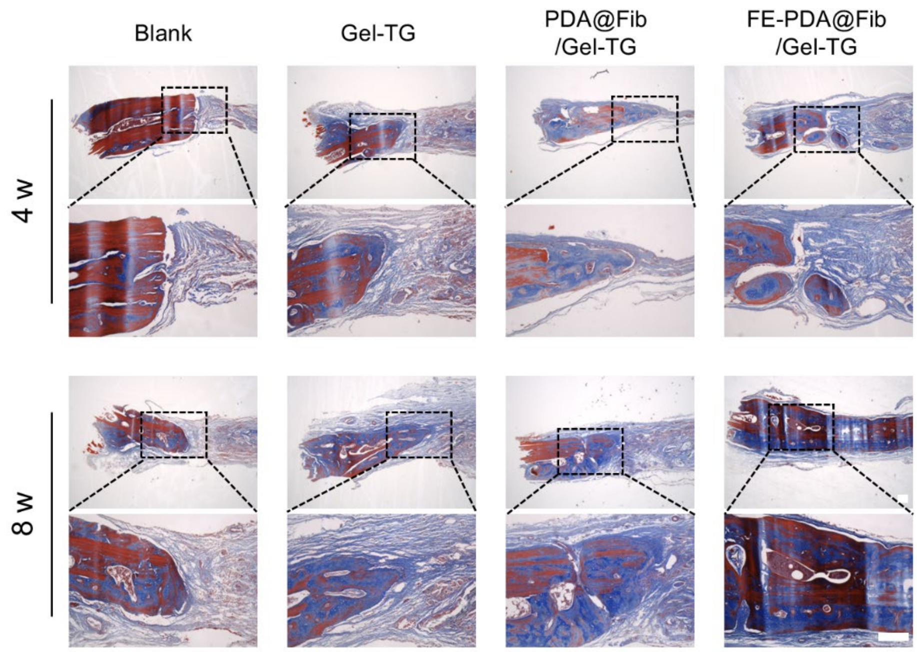

Figure S17. Masson's trichrome staining of explants harvested 4 and 8 weeks after implantation, showing collagen deposition and bone matrix maturation. Scale bar: 200 μm.
